# A translational preclinical strategy for chronic spinal cord injury: neuroprotective and regenerative potential of botulinum neurotoxin type A combined with muscle atrophy prevention via electrostimulation

**DOI:** 10.1016/j.mmr.2026.100049

**Published:** 2026-06-27

**Authors:** Valentina Mastrorilli, Siro Luvisetto, Veronica Ruggieri, Giada Raparelli, Luca Madaro, Lucia Amalia Paggi, Chiara Parisi, Francesca De Santa, Federica De Angelis, Annunziata D’Elia, Roberto Massari, Susanna Amadio, Ornella Rossetto, Valentina Vacca, Maurizia Caruso, Gianluca Sferrazza, Flaminia Pavone, Sara Marinelli

**Affiliations:** a National Research Council of Italy, Institute of Biochemistry and Cell Biology, 00015 Monterotondo (RM), Italy; b Department of Anatomical, Histological, Forensic Sciences and Orthopedics, Sapienza University of Rome, 00161 Rome, Italy; c Laboratory affiliated to Istituto Pasteur Italia-Fondazione Cenci Bolognetti, 00161 Rome, Italy; d Department of Biological, Geological, and Environmental Sciences, Alma Mater Studiorum University of Bologna, 40126 Bologna, Italy; e Cellular Neurobiology Unit, Santa Lucia Foundation, 00179 Rome, Italy; f Department of Biomedical Sciences, University of Padova and National Research Council of Italy, Institute of Neuroscience, 35131 Padova, Italy; g Department of Biomedical Science, National Research Council of Italy, 00185 Rome, Italy

**Keywords:** Spinal cord injury (SCI), Botulinum neurotoxin type A (BoNT/A), Electrical muscle stimulation, Neuroglia, Remyelination, Neuropathic pain

## Abstract

**Background:**

Spinal cord injury (SCI) triggers persistent neuroinflammation, gliosis, neuronal loss, and demyelination, leading to motor deficits and neuropathic pain (NeP). Botulinum neurotoxin type A (BoNT/A) has shown anti-inflammatory and neuroprotective effects in acute SCI, but its potential in the chronic phase remains unclear. This study investigates whether combining BoNT/A with electrical muscle stimulation (EMS) enhances recovery in chronic SCI.

**Methods:**

Adult mice with severe thoracic SCI (paraplegic) underwent EMS (30 min/d for 10 non-consecutive days starting 3 d post-injury) or no stimulation. Fifteen days after SCI, animals received a single intrathecal injection of BoNT/A (15 pg/5 μl) or saline. Functional recovery was assessed up to 60 d as well as in moderate and mild SCI mice. NeP onset and maintenance were evaluated. Spinal cord tissue was analysed for astrocytic and microglial morphology, neuronal and oligodendroglial survival, myelin protein expression, and *in vitro* effects on oligodendrocyte precursor cells (OPCs). The phenotype of hindlimb muscles was evaluated through morphological and gene expression analyses.

**Results:**

EMS was able to counteract muscle atrophy and fibrosis, and when combined with BoNT/A, also denervation. Moreover, the combination restored hindlimb motor function in chronic SCI, whereas BoNT/A or EMS alone were ineffective. NeP, a common comorbidity associated with SCI, was mitigated by BoNT/A treatment even when administered in the chronic phase. BoNT/A reduced astrocytic hypertrophy and excitatory synapse association and was associated with a morphology-based redistribution of microglial profiles toward a resting-like classification, decreased apoptosis, and increased neuronal and oligodendroglial survival. Myelin basic protein (MBP) expression was significantly elevated *in vivo*. *In vitro*, BoNT/A promoted OPC differentiation into myelinating oligodendrocytes, increased process complexity, and upregulated MBP, galactocerebroside C, proteolipid protein, and myelin oligodendrocyte glycoprotein under both proliferative and differentiating conditions. Cleaved synaptosomal-associated protein 25 colocalization with OPC confirmed direct BoNT/A internalization and activity.

**Conclusions:**

BoNT/A exerts neuroprotective effects in chronic SCI by reducing neuroinflammation and supporting neuronal and oligodendroglial preservation. When combined with EMS, it also promotes remyelination and improves muscle homeostasis, suggesting that early stimulation creates a permissive environment for recovery. These findings support the clinical evaluation of BoNT/A as a therapeutic strategy for chronic SCI.

## 1 Background

Traumatic spinal cord injuries (SCI) represent a significant and growing public health challenge, with an estimated 0.9 million new cases reported annually and over 20 million people globally affected by SCI-related disabilities [1]. These injuries commonly result from domestic accidents, occupational hazards, vehicular accidents, sports-related trauma, and interpersonal violence, leading to a spectrum of sensory and motor dysfunctions. Epidemiological analyses confirm that SCI disproportionately affects young adults, primarily men between 30 and 40 years of age, resulting in a long survival window during which chronic symptoms accumulate and quality of life is profoundly impacted [1], [2], [3]. Beyond these primary impairments, SCI is associated with various complications, such as neuropathic pain (NeP) affecting up to 80% of patients, gastrointestinal issues, spasticity, and reduced life expectancy, which collectively create a substantial economic burden, exceeding $4 billion annually in costs related to home care, adaptive housing, medical visits, and hospitalizations (United States estimation) [2], [3]. Given this impact, there is an urgent need for therapies that can address not only the initial injury but also the long-term disability associated with SCI.

Regenerative medicine has made notable advances in treating SCI through neuroprotective agents, genetic interventions, and pro-regenerative biotechnologies [4]. However, central nervous system (CNS) injuries remain highly resistant to repair due to the complex structural and molecular characteristics of the CNS, particularly the inhibitory environment created by severe injuries like SCI. After SCI, vascular disruption, ischemia, and inflammation hinder axonal regrowth, while the formation of a glial scar serves as a physical and biochemical barrier to repair [5]. This regenerative failure results from the lack of growth-promoting signals and the accumulation of inhibitory factors at the injury site, which together prevent neuronal recovery.

SCI progresses through acute, subacute, and chronic phases, with each stage presenting distinct therapeutic challenges. While early interventions may hold some potential during the acute phase, the chronic phase, beginning approximately 4 weeks post-injury, is marked by more profound and often irreversible losses in motor and sensory function. Chronic SCI is characterized by persistent neuroinflammation, glial scar formation, and muscle atrophy, all of which complicate any therapeutic approach [6], [7]. This inflammatory response, although useful for initial clearance of debris, can exacerbate tissue damage mediated by proinflammatory cytokines from activated microglia and infiltrating immune cells. Moreover, the degeneration of oligodendrocytes and subsequent demyelination further degrade the white matter, worsening functional outcomes [6], [7].

In this context, botulinum neurotoxin type A (BoNT/A) stands out as a promising candidate for SCI treatment due to its unique biochemical and therapeutic properties. BoNT/A is a neurotoxin that specifically cleaves synaptosomal-associated protein 25 (SNAP25) [8], a crucial soluble NSF attachment protein receptor (SNARE) protein essential for neuroexocytosis [9]. By blocking synaptic vesicle release, BoNT/A effectively modulates the secretion of neurotransmitters and inflammatory molecules, making it a valuable therapeutic agent across a spectrum of over 100 conditions, ranging from muscular to neurological disorders [10]. Widely utilized in clinical SCI settings for its efficacy in treating spasticity, neurogenic bladder dysfunction, and NeP, BoNT/A has demonstrated a favourable safety profile with well-defined and manageable adverse effects, contributing to its reputation as a safe and effective treatment.

One of BoNT/A’s most beneficial attributes in clinical practice is its prolonged duration of action, often lasting several months due to its biochemical mechanism, which enables long-term effects from a single administration [11]. This prolonged action supports sustained symptom relief, reducing the need for frequent treatments and improving patient compliance. Additionally, several studies have uncovered broader pharmacological effects of BoNT/A, revealing theanti-inflammatory and neuroprotective properties that make it an appealing candidate for neuroinflammatory conditions [12]. In preclinical SCI models, we have shown that BoNT/A administration during the acute phase [13], [14] can promote motor recovery, decrease inflammation, and support axonal repair. These findings highlight its capacity to positively modulate the CNS environment, offering benefits beyond its established role in muscle tone and spasticity management. Given the high prevalence of SCI and the extensive demands of chronic care, we now focus on the therapeutic application of BoNT/A in the chronic phase of SCI, where intervention options remain extremely limited. For this purpose, we developed a murine model that mimics the chronic aspects of human SCI, including sustained neuroinflammation, muscle atrophy, and sensory-motor deficits [15], [16], together with muscle rehabilitation with electrostimulation to mitigate atrophy.

Our aim was to test the BoNT/A’s transformative role in neuroinflammation and remyelination and also to pave the way for translating preclinical findings into clinical practice. Indeed, the present study is part of a repositioning pharmacological research and development program aimed at identifying new mechanisms of action of BoNT/A and a new route of drug administration, promoting its future use as an innovative experimental medicine for the treatment of patients with SCI [17], by a new multidisciplinary approach to the management of SCI, and thus improving their quality of life.

## 2 Methods

### 2.1 Animals

Four to six-month-old CD1 female mice (*n*=135, EMMA Infrafrontier, Monterotondo, Italy) were used. Weighing 35–40 g at the beginning of the experiments, mice were housed in groups of 4 or 8 in standard cages under a 12 h/12 h light/dark cycle (7:00 a.m.–7:00 p.m.) with food and water available ad libitum. Before being used for any experimental setup, the animals were acclimated in our animal facility for at least 30 d and, similarly, 30 min before surgery, they were placed in the experimental room for acclimatization. Testing was done by blind investigators for the treatment groups (as better described in **Additional file 1: Methods**). Care and handling of mice were in accordance with the guidelines of the Committee for Research and Ethical Issues of IASP (PAIN® 1983, 16, 109–110). The experimental protocol for *in vivo* procedures was approved by the Italian Ministry of Health (protocol code 122/2019PR). The number of animals utilized for each experiment and group is reported in Table 1.

**Table 1:** Experimental groups.

**Assay**	**Groups**
Basso Mouse Score	SCI (*n*=6); EMS+saline (*n*=10); EMS+BoNT/A (*n*=16)
Aesthesiometer/Plantar (neuropathic pain)	EMS+saline (*n*=9); EMS+BoNT/A (*n*=10)
Muscle atrophy and neuromuscular junction	Naive (*n*≥4); SCI (*n*≥4); EMS+saline (*n*≥4); EMS+BonT/A (*n*≥4)
Immunofluorescences	SCI (*n*=2–4); EMS+saline (*n*≥3); EMS+BonT/A (*n*≥3)
Western blotting	Naive (*n*=6); SCI (*n*=4); EMS+saline (*n*=7); EMS+BonT/A (*n*=4)

SCI. Spinal cord injury; EMS. Electrical muscle stimulation; BoNT/A. Botulinum toxin serotype A

### 2.2 Surgery

**Fig. 1:**
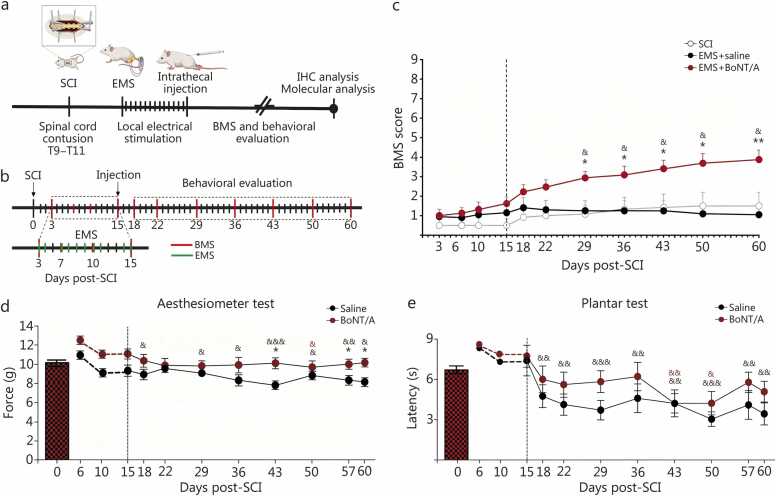
Effects of combination of EMS and BoNT/A on motor recovery. **a** Schematic representation of the experimental paradigm combining a rehabilitative protocol with spinal BoNT/A injection. The study design integrates an EMS protocol, aimed at preventing muscle atrophy, with a single intrathecal injection of BoNT/A administered 15 d after spinal cord injury (SCI). **b** Detailed timeline of the EMS protocol, with green line indicating the scheduled days of EMS. Red lines mark the days on which behavioral assessments were performed. **c** Basso Mouse Scale (BMS) evaluation of motor function in mice with severe SCI (BMS score 0–3). Groups included: untreated SCI animals (SCI), animals receiving EMS combined with intrathecal saline injection 15 d post-injury (EMS+saline), and animals receiving EMS combined with BoNT/A (EMS+BoNT/A). Dotted line indicates the day of treatment. No differences before treatment were revealed between groups. After treatment (from day 18) repeated measures ANOVA revealed a significant effect of treatment (*F*_2,29_=6.137, *P=*0.0006), time (*F*_6,12_=4.529, *P=*0.0003), and treatment × time interaction (*F*_12,174_=4.431, *P*<0.0001). Tukey-Kramer post hoc analysis showed significant differences at D29, D36, D43, D50, and D60 between EMS+BoNT/A vs. SCI groups (⁎*P*<0.05, ⁎⁎*P*<0.005) and EMS+BoNT/A vs. EMS+saline groups (^&^*P*<0.05, ^& &^*P*<0.005) (SCI: *n*=6; EMS^+^saline: *n*=10; EMS*+*BoNT/A: *n*=16). **d** Mechanical allodynia was assessed in mildly to moderately contused mice using the Dynamic Plantar Aesthesiometer (force expressed in grams). A repeated measures ANOVA conducted between D18 and D60 post-SCI revealed a significant main effect of treatment (*F*_1,34=_8.278, *P=*0.0069), with no significant effects for time or for the treatment × time interaction. Each group included 9 animals (*n*=18 values per time point, considering both hind paws were analyzed separately). No significant differences were detected between groups or for the group × time interaction during the pre-treatment phase (repeated measures ANOVA). Post hoc Tukey-Kramer analysis revealed significant differences between BoNT/A- and saline-treated mice at D43, D57, and D60 (⁎*P<*0.05). Paired *t*-tests vs. baseline (D0, pre-SCI) (^&^*P*<0.05, ^& &^*P*<0.005, ^& & &^*P*<0.0001; ^&^ in black indicates the saline group, in red indicates the BoNT/A group) showed a significant reduction in mechanical threshold in BoNT/A-treated mice at D50 (*t*₁₇=2.122, *P=*0.048), whereas saline-treated mice displayed a significant development of mechanical allodynia at D18 (*t*₁₇=2.11, *P=*0.0500), D29 (*t*₁₇=2.297, *P=*0.0340), D36 (*t*₁₇=3.017, *P=*0.0078), D43 (*t*₁₇=5.773, *P<*0.0001), D50 (*t*₁₇=2.198, *P=*0.0421), D57 (*t*₁₇=4.041, *P=*0.0008), and D60 (*t*₁₇=2.97, *P=*0.0086). **e** Thermal hyperalgesia was evaluated in mildly to moderately contused mice using the Plan*t*ar test. During the pre-treatment phase, no significant differences were observed between groups or with respect to baseline values. Repeated measures ANOVA conducted for the post-treatment period (D18–D60) did not reveal significant effects for treatment, time, or treatment × time interaction, although treatment approached statistical significance (*F*_1,36=_3.539, *P=*0.0680). Paired *t-*tests vs. baseline (D0, pre-SCI) (^&^*P*<0.05, ^& &^*P*<0.005, ^& & &^*P*<0.0001; ^&^ in black indicates the saline group, in red indicates the BoNT/A group) showed a significant decrease in thermal withdrawal latency (indicative of thermal hyperalgesia) in BoNT/A-treated mice at D43 (*t*₁₉=3.175, *P=*0.005) and D50 (*t*₁₉=2.182, *P=*0.0419). In saline-treated animals, significant thermal hyperalgesia compared to baseline was observed at D18 (*t*₁₇=3.929, *P=*0.0011), D22 (*t*₁₇=4.682, *P=*0.0002), D29 (*t*₁₇=5.420, *P<*0.0001), D36 (*t*₁₇=2.906, *P=*0.0035), D43 (*t*₁₇=4.577, *P=*0.0003), D50 (*t*₁₇=6.730, *P<*0.0001), D57 (*t*₁₇=4.750, *P=*0.0002), and D60 (*t*₁₇=4.720, *P=*0.0002). Each time point included 20 measurements for BoNT/A (10 animals × 2 hind paws) and 18 for saline (9 animals × 2 hind paws). EMS. Electrical muscle stimulation; BoNT/A. Botulinum neurotoxin type A.

To induce severe SCI (experimental design Fig. 1**a**), animals were deeply anesthetized using either a 1:1 mixture of Rompun (Bayer, 20 mg/ml; 0.5 ml/kg) and Zoletil (100 mg/ml; 0.5 ml/kg), or connected to an inhalation anaesthesia system delivering isoflurane (1%–3%) in a continuous oxygen flow (2 L/min) through a nose cone. After shaving the back with an electric razor, the skin was disinfected with Betadine and incised to expose the vertebral column [13], [14]. Animals were then secured in a stereotaxic frame equipped with spinal adaptors and connected to the PinPoint Precision Impactor Device (Stoelting; Hatteras Instruments Inc., Cary, NC, USA). Core body temperature was maintained at 37 ℃ throughout the procedure.

Spinal cord contusion was induced without laminectomy at thoracic levels T9–T11 (**Additional file 1:** Fig. S1), using the following parameters: impactor tip #4 (middle, round, flat), velocity 3 m/s, depth 5 mm, and dwell time 800 ms. Although most experiments were conducted on animals with severe injuries [Basso Mouse Scale (BMS) [18] score 0–3], a subset of animals with moderate (BMS score 4–6) or mild (BMS score 7–9) injuries was also included in specific behavioral assays to evaluate the potential effects of treatment on NeP. Following surgery, animals were monitored daily for postoperative complications, as explained in **Additional file 1: Methods**.

### 2.3 Electrical muscle stimulation

As part of the rehabilitation strategy, we initially explored a passive treadmill-based paradigm (**Additional file 1: Methods** and Fig. S2) to engage residual spinal locomotor circuits (central pattern generators) [19]. Seventy-two hours after surgery, animals were randomly assigned to stimulated or non-stimulated groups. Mice in the stimulated group underwent hind paw electrical muscle stimulation (EMS) for 30 min per session, across 10 non-consecutive days (EMS protocol Fig. 1**b**).

Animals undergoing EMS were classified as “severe” SCI cases, defined by complete hindlimb paralysis (BMS scores 0–3, where 0 indicates no movement and 9 indicates full motor recovery). Animals with BMS scores between 4 and 7, classified as “moderate” or “mild”, did not receive EMS but were included in the study to evaluate the toxin’s impact on NeP during the chronic phase. Neuropathic assessments included responses to non-painful mechanical stimuli (dynamic aesthesiometer plantar test for allodynia), and painful thermal stimuli (plantar test for hyperalgesia). Given the variability in neuropathy onset between limbs, each paw was evaluated independently.

Stimulation parameters were: pulse duration 160 μs, frequency 60 Hz, delivered using a Tesmed® stimulator (Shenzhen Roundwhale Technology Co., Ltd., Shenzen, China), similar to protocols utilized in murine models [20]. The smallest (4 cm×4 cm) transcutaneous electrode patches were utilized, and the hindlimbs were entirely enveloped (**Additional file 2: Video S1**). To optimize electrical conductivity, fur was shaved from the hind paws, and an electroconductive gel was applied prior to each session. Stimulation was performed in a dedicated experimental cage where animals were temporarily housed and allowed to move freely; no physical restraint was required. Only mice with severe SCI were included in the EMS group. Accordingly, only a minimal number of severely injured animals (*n*=6) were assigned to the non-stimulated control group, in compliance with the 3Rs principles and based on previously published evidence of absent spontaneous recovery in this model (**Additional file 1: Methods**) [13], [14], [15].

Supplementary material related to this article can be found online at doi:10.1016/j.mmr.2026.100049.

The following is the Supplementary material related to this article Video S1.

Video S1

### 2.4 Drug administration

Fifteen days after SCI, animals were re-anesthetized using inhalation anaesthesia as previously described. A single intrathecal injection of either BoNT/A (15 pg/5 μl; 150 kD purified neurotoxin, gently gifted by Prof. C. Montecucco, University of Padua) or saline (0.9%, 5 μl) was administered using a 10 μl Hamilton® syringe (30-gauge needle; Biosigma, Cona, VE, Italy).

As previously demonstrated, BoNT/A is capable of retrograde axonal transport [12], [21], [22], [23]. To minimize the risk of systemic diffusion potentially associated with ischemic events at the site of contusion, the injection was performed caudally, between the lumbar vertebrae L4 and L5.

### 2.5 High-resolution *in vivo* micro-computed tomography imaging for quantitative analysis of spinal skeletal structures

Micro-computed tomography (micro-CT, **Additional file 1:** Fig. S1) was employed to obtain high-resolution *in vivo* datasets of spinal skeletal morphology using a MILabs Hybrid OI/CT imaging system (MILabs, Houten, The Netherlands). Mice were anesthetized via inhalation of isoflurane (1%–3%) in a continuous oxygen flow (2 L/min) delivered through a nose cone, ensuring stable immobilization during image acquisition. Scans were performed in accurate acquisition mode, comprising 720 angular projections over a 360° rotation arc. The X-ray tube was set to 35 kV (0.43 mA), with an exposure time of 200 ms per projection. All acquisition parameters were held constant across experimental subjects to ensure reproducibility and facilitate comparative analysis. No respiratory or cardiac gating was applied. Each imaging session lasted approximately 5 min, generating volumetric datasets with high isotropic resolution suitable for detailed structural evaluation of vertebral components. Three-dimensional skeletal reconstruction is detailed explained in **Additional file 1: Methods**.

### 2.6 Behavioral tests

For injured animals, the hindlimb locomotor function was assessed in an open field. The BMS score ranges from 0 to 9, where 0 indicates complete paralysis and 9 normal movements of the hindlimbs [18]. Performances of the left and right hindlimbs were averaged to obtain the BMS score. Mice were tested for hindlimb functional deficits at D3, D6, D10, D15 (injection timepoint), D18, D22, D29, D36, D43, D50, D57, and D60 after SCI. Severely, moderately, and mildly injured mice were included in this test. Mild and moderate contused animals were assigned to NeP evaluation tests.

Based on preliminary experiments showing that BoNT/A administered alone in the chronic phase did not restore motor function in this severe model (**Additional file 1:** Fig. S3), subsequent mechanistic analyses were focused on EMS-conditioned cohorts to evaluate therapeutic efficacy within a functionally permissive context.

### 2.7 Mechanical and thermal allodynia: Dynamic Plantar Aesthesiometer and plantar test

Mechanical allodynia was tested using a Dynamic Plantar Aesthesiometer (Model 37400, Ugo Basile Srl, Comerio, Italy) as previously described [24].

For habituation, mice were placed 30’ before the test in the experimental room and in testing plastic cages with a wire net floor 5–10 min before the experiment. Each testing day, the withdrawal threshold of hind paws was taken as the mean of three consecutive measurements per paw.

Thermal hyperalgesia was tested using an automatic plantar test instrument (Plantar Test, Ugo Basile, Comerio, Italy). For the thermal hyperalgesia test, a cut-off time of 15 s was imposed to avoid damage to the hind paw skin tissue.

Mechanical and thermal threshold were measured at D3, D6, D10, D15 (injection timepoint), D18, D22, D29, D36, D43, D50, D57, and D60 after SCI and tests were conducted 1 h spaced-apart. For each mouse, two values of mechanical and thermal threshold were obtained because the two hind paws, the right and the left, can develop different degrees of NeP. At each testing day, threshold values were averaged from three consecutive measurements per hind paw.

### 2.8 Histological staining

#### 2.8.1 Confocal microscopy and quantification of immunoresponsivity

Immunostained sagittal spinal cord sections were imaged using a TCS SP5 laser scanning confocal microscope (Leica Microsystems, Buccinasco, MI, Italy). All acquisitions were performed in sequential scanning mode to eliminate cross-channel bleed-through. Both low (10× objectives) and high (40× and 63× objectives) magnification images were acquired and processed using I.A.S. software (Delta Systems, Italy). Although 63× images were also acquired and analyzed, only 40× images were selected as representative images to ensure consistency and homogeneity of presentation throughout the manuscript. Quantitative analyses were carried out with Fiji/ImageJ software (National Institutes of Health, USA). Quantitative analyses included automated cell counting, area-based morphological measurements, and fluorescence intensity quantification; detailed protocols, segmentation parameters, and magnification correction procedures are reported in the **Additional file 1: Methods** and Fig. S3.

#### 2.8.2 Morphometric and Sholl analysis

Morphometric analysis was performed on high-resolution images acquired using the following confocal parameters: 40× or 63× objective, 3× digital zoom, 1024×1024 frame size, and 10 Hz scanning speed. Each image was converted to binary format to generate digital silhouettes of astrocytes or microglial cells, allowing for the identification and measurement of cellular outlines. Activated microglia were quantified using a previously established classification method [13].

Briefly, based on morphological features such as circularity and process length, microglia were categorized into distinct activation states: ramified (resting microglia), hyper-ramified (bushy or hyperactive microglia), and unramified/amoeboid (fully activated microglia). Cell classification was guided by representative reference images for each phenotype.

Sholl analysis was conducted on ionized calcium binding adaptor moleclule 1 (Iba1)⁺/cluster of differentiation 11b (Cd11b)^+^ microglia, glial fibrillary acidic protein (GFAP)⁺ astrocytes, and differentiated oligodendrocyte precursor cells (OPCs) to assess the complexity of process arborization (quantified as the number of intersections between microglial processes and concentric circles at increasing radial distances from the soma). Cell diameter was defined as the maximum radial distance from the soma to the tip of the longest projection. Concentric circles were drawn at 5 μm intervals from the cell soma, and the number of intersections per radius was quantified. This analysis enabled the evaluation of branching complexity as a function of radial distance from the cell body, following the method described by Paes-Colli *et al*. [25]. Quantification was performed using the Sholl Analysis plugin in Fiji/ImageJ (NIH, Bethesda, MD, USA; developed by Wayne Rasband), according to the developer’s instructions.

These data were used to evaluate microglial, astrocytic, and OPC process complexity and branching patterns in response to SCI.

#### 2.8.3 *In vitro* oligodendrocyte cell cultures and BoNT/A treatment

Rat glial precursor cells (GPCs), also referred to as OPCs (Gibco; Thermo Fisher Scientific, Whaltam MA, USA; N7746100) were thawed and seeded at a concentration ranging from 1×10^5^ to 2.5×10^5^ cells/cm^2^ in complete OPC growth medium [KnockOut™ D-MEM™/F-12, 2% StemPro™ NSC SFM™ supplement, 20 ng/ml epidermal growth factor (EGF), 20 ng/ml basic fibroblast growth factor (bFGF), 2 mmol/L GlutaMAX™-I and 10 ng/ml platelet derived growth factor-AA (PDGF-AA) on poly-L-ornithine (20 μg/ml) coated tissue culture plates, or coverslips].

Complete OPC medium was changed every two days to maintain undifferentiated proliferating cells. Otherwise, to induce spontaneous oligodendrocyte differentiation and maturation, OPC medium was replaced with oligodendrocyte differentiation medium [KnockOut™ D-MEM™/F-12, 2% StemPro™ NSC SFM™ supplement, 2% fetal bovine serum (FBS, 2 mmol/L GlutaMAX™-I) and replaced with fresh medium every day].

To characterize the responsiveness of OPCs to BoNT/A, OPCs and differentiating oligodendrocytes were treated with vehicle or BoNT/A (10 pmol/L) for 48 h.

To assess cell viability, cells were detached using StemPro™ Accutase™ Cell Dissociation Reagent (Gibco; Thermo Fisher Scientific, Whaltam MA, USA), stained with trypan blue and counted using a hemocytometer.

For detailed methods of RNA analysis, muscle immunofluorescence staining, Sirius red staining, and so on are described in **Additional file 1: Methods** and Tables S1-S2**.**

### 2.9 Statistical analysis

The group size for *in vivo* experiments was calculated by implementing a power analysis (Gpower 3.1). For locomotor recovery (BMS score; 11 repeated measures across three groups), sample size was calculated assuming effect size *f*=0.25, α=0.05, 1–β=0.95 and indicated that 27 animals were required. For NeP tests (aesthesiometer and plantar test), repeated-measures ANOVA was performed separately for pre-treatment (4 measures, including baseline) and post-treatment (8 measures). Power analyses with effect size *f*=0.25, α=0.05, 1–β=0.80 yielded required sample sizes of 24 (pre-treatment) and 16 (post-treatment). The number of mice used is reported in the figure legends. With regard to immune and biochemical experiments, the sample size was estimated according to previous experience, using the models described. Experimental data were expressed as mean±standard error of the mean (SEM). Group comparisons were conducted by one-way or two-way ANOVA for repeated measures or by Student’s *t*-test. Post hoc comparisons were made with Tukey-Kramer tests (statistical significance at *P*<0.05). Data analysis was performed by StatView SAS (version 5.0, Cary, NC, USA) or Prism GraphPad (San Diego, CA, USA).

For datasets with a limited number of animals (*n*<5 per group), or when normality and homogeneity of variance could not be assumed, non-parametric tests were applied. Comparisons between two independent groups were performed using the Mann-Whitney *U* test (also referred to as the Wilcoxon rank-sum test). For analyses involving three or more independent groups, the Kruskal-Wallis test followed by Dunn’s post hoc test (with Holm correction for multiple comparisons) was used to identify pairwise differences. To assess the effect of two independent factors (e.g., treatment and area) and their potential interaction, we used the Scheirer-Ray-Hare test (SRH), a rank-based, non-parametric alternative to the two-way ANOVA. This method, implemented in both R and Python, extends the Friedman/Kruskal-Wallis approach to multifactorial designs, allowing analysis of main effects and interactions without assuming data normality.

Data are reported as median [interquartile range (IQR)], with individual animal values plotted for each group. All analyses were conducted considering the animal as the experimental unit, with slice-level data averaged per animal to avoid pseudoreplication.

## 3 Results

### 3.1 Differential effects of BoNT/A in severe and moderate SCI: combining rehabilitation for motor recovery and pain management

Our initial goal was to investigate whether intrathecal administration of BoNT/A during the onset of the chronic phase in paraplegic mice could restore motor function, as previously demonstrated when administered in the acute phase [13], [14]. Mice subjected to a severe thoracic SCI (T9–T11; see **Additional file 1:** Fig. S1), as described in our earlier work [15], received a single intrathecal injection of either saline or BoNT/A (15 pg/5 μl) at the lumbar level (L5). This injection site was chosen to minimize the risk of systemic toxin spread, particularly relevant in ischemic regions near the lesion, and to exploit the known retrograde transport capacity of BoNT/A to reach distant targets [12], [21], [26]. However, motor function assessed using the BMS score [18] showed that BoNT/A alone, administered during the chronic phase, did not produce functional recovery, despite detectable central enzymatic activity (**Additional file 1:** Fig. S4**)**, indicating that central modulation in isolation is insufficient in the presence of severe muscle atrophy.

These results led us to re-examine earlier studies [13], [15], [16], demonstrating that paraplegic animals experience pronounced muscle atrophy as early as D7 post-trauma. Based on this, we hypothesized that the evaluation of BoNT/A’s effects, when administered in the chronic phase, required a rehabilitative approach to mitigate muscle atrophy. To address this, we developed a hindlimb muscle electrostimulation (EMS) protocol using transcutaneous electrodes to deliver electrical stimulation from D3 to D15 post-trauma (protocol illustrated in Fig. 1**a** and **Additional file 2: Video S1**). On D15, animals were randomly divided into a control group (vehicle: saline) and a treatment group (BoNT/A), with EMS discontinued at this stage. Motor recovery was assessed throughout the EMS phase and after treatment (Fig. 1**b**).

Fig. 1**c** illustrates that severe SCI animals undergoing EMS rehabilitation showed no significant motor improvement up to D15 post-lesion compared to non-EMS-treated SCI animals (all detailed statistical analyses are included in legend text). However, when BoNT/A or saline was spinally administered at D15, and EMS was discontinued, only the group EMS+BoNT/Aexhibited gradual and significant motor recovery, reaching approximately 50% recovery in motor performance (**Additional file 3: Video S2**).

Supplementary material related to this article can be found online at doi:10.1016/j.mmr.2026.100049.

The following is the Supplementary material related to this article Video S2.

Video S2

Regarding BoNT/A’s ability to mitigate NeP during the chronic phase, moderate and mild SCI animals showed significant prevention of allodynia compared to saline-treated SCI mice (Fig. 1**d**). Additionally, while both groups developed hyperalgesia, BoNT/A treatment significantly reduced pain levels compared to saline-treated controls (Fig. 1**e**).

### 3.2 EMS in the post-acute phase of SCI: a rehabilitation strategy to prevent muscle atrophy and facilitate BoNT/A regenerative action

**Fig. 2:**
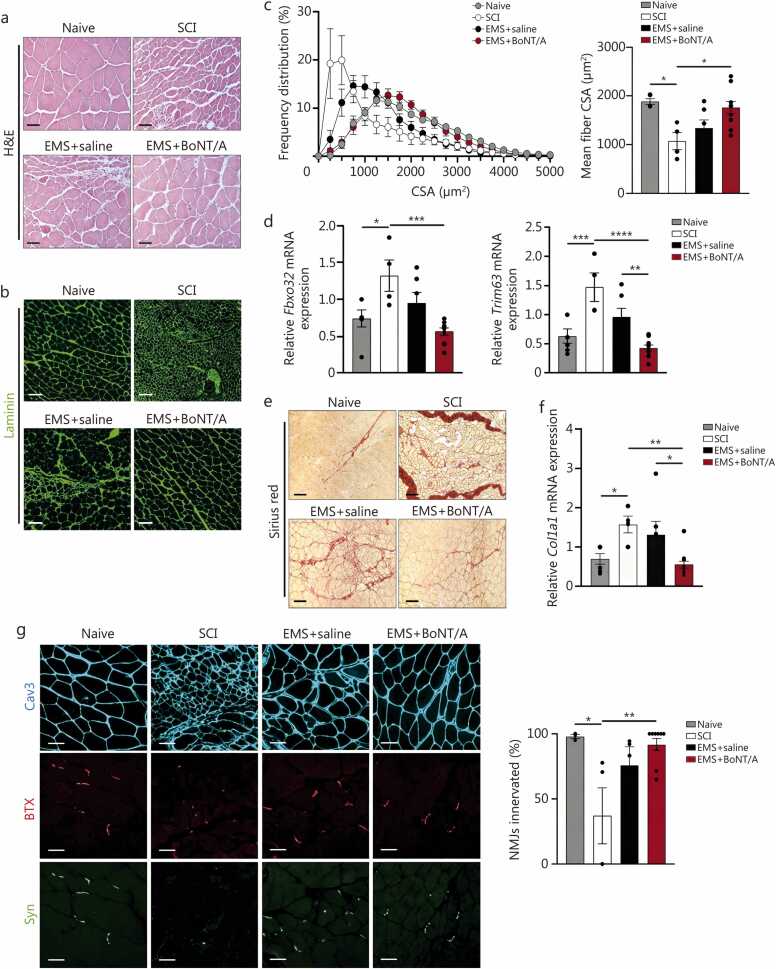
EMS and BoNT/A prevent muscle atrophy and preserve neuromuscular integrity in chronic SCI. **a** Representative micrographs of gastrocnemius (GA) muscle sections from naive, SCI, EMS+saline, and EMS+BoNTA mice stained with haematoxylin and eosin (H&E). Scale bar=100 μm. **b** Representative immunostaining for Laminin (green) in GA muscle sections from naive, SCI, EMS+saline, and EMS+BoNTA mice. Scale bar=100 μm. **c** Frequency distribution of myofiber cross-sectional area (CSA) in gastrocnemius muscles from naive, SCI, EMS+saline, and EMS+BoNT/A mice (left). Data were analyzed by two-way ANOVA (CSA class × treatment), revealing a significant effect of CSA class (*F*_20,441_=38.63, *P*<0.0001) and a significant interaction between CSA class and treatment (*F*_60,441=6.439_, *P*<0.0001), whereas the overall treatment effect was not significant (*F*_3,441_=0.00149, *P*>0.9999). Tukey’s post hoc analysis showed that EMS+BoNT/A mice differed significantly from SCI animals at CSA classes ranging from 250 to 2250 µm² (*P*<0.05–0.0001), whereas differences between EMS+BoNT/A and EMS+saline mice were detected at 500, 750, 1000, 1750 and 2000 µm² (*P*<0.05–0.0001). No significant differences were observed between EMS+BoNT/A and naive mice across the CSA distribution. Mean of myofiber CSA measured on sections of GA muscle from naive, SCI, EMS+saline, and EMS+BoNTA mice (*n*≥3, right). Values represent mean±SEM. Statistical sig*n*ificance was assessed by one-way ANOVA test: *F*_3,21_=5.752, *P=*0.0049. Post hoc tests for multiple comparisons between groups were performed by Tukey-Kramer test: SCI vs. naive *P*<0.05, EMS+BoNT/A vs. SCI *P*<0.05. **d** Relative mRNA expression of *Fbxo32* and *Trim63* genes in GA muscle from naive, SCI, EMS+saline, and EMS+BoNTA mice (*n*≥4 for each groups). Values represent mean±SEM. One-way ANOVA test: *F*_3,23_=7.690, *P=*0.0010; *F*_3,24_=14.23, *P*<0.0001, respectively. Post hoc tests for multiple comparisons between groups were performed with Tukey-Kramer test. **e** Representative micrographs of GA muscle sections from naive, SCI, EMS+saline, and EMS+BoNTA mice stained with Sirius red. Scale bar=100 μm. **f** The relative mRNA expression of *Col1a1* gene in GA muscle from the same groups (*n*≥4). Values represent mean±SEM. One-way ANOVA test: *F*_3,24_=6.474, *P=*0.0023. Post hoc tests for multiple comparisons between groups were performed with Tukey-Kramer test. **g** Representative immunostaining for caveolin-3 (Cav3) (cyan) and synaptophysin (Syn) (green) on GA muscle sections from naive, SCI, EMS+saline and EMS+BoNTA mice. Neuromuscular junctions (NMJs) are labelled with fluorescent alpha-bungarotoxin (BTX) (red). Scale bar=50 μm. The graph on the right shows the percentage of innervated NMJs calculated by quantifying the Syn and BTX overlapping signals (*n*≥3). Values represent mean±SEM. One-way ANOVA test: *F*_3,16_=5.315, *P=*0.0098. Post hoc tests for multiple comparisons between groups were performed with Tukey-Kramer test. ⁎*P<*0.05, ⁎⁎*P<*0.01, ⁎⁎⁎*P<*0.001, ⁎⁎⁎⁎*P<*0.0001. EMS. Electrical muscle stimulation; BoNT/A. Botulinum neurotoxin type A; SCI. Spinal cord injury; Fbxo32*.* F-box protein 32 (also known as MAFbx/Atrogin-1); Trim63. Tripartite motif containing 63 (MuRF1); SEM. Standard error of the mean.

Building on our previous findings [13], [15], [16], which demonstrated rapid muscle decline and atrophy as significant barriers to evaluating regenerative therapies in SCI, we considered clinical evidence suggesting that EMS in individuals with SCI can offer various benefits [27]. These include increased muscle and bone mass, reduced fat mass, decreased spasticity, and improved functional mobility [28]. To address hindlimb muscle atrophy in our severe SCI model, we implemented EMS prior to BoNT/A treatment to assess its potential in preventing or mitigating muscle loss. SCI animals underwent the EMS protocol illustrated in Fig. 1**a** and **Additional file 2: Video S1**. After 60 d, gastrocnemius (GA) muscles from different experimental groups (naive, SCI, EMS+saline, and EMS+BoNT/A) were collected and analysed (Fig. 2).

Morphological analyses revealed that GA muscles from SCI-subjected mice exhibited a marked reduction in fibre size, quantified as a decrease in cross-sectional area (CSA) (Fig. 2**a-c**). Specifically, SCI mice showed a significant shift toward a higher frequency of smaller fibres together with a significant reduction in mean fibre CSA compared to naive controls. EMS treatment alone induced only a partial recovery profile, whereas the EMS+BoNT/A group showed a significant restoration of muscle morphology compared with SCI animals, reaching values no longer significantly different from naive mice (Fig. 2**c**).

We also examined the mRNA expression levels of F-box protein 32 (*Fbxo32*) [29] (also known as *MAFbx/Atrogin-1*) and tripartite motif containing 63 (*Trim63*) [30] (*MuRF1*), two muscle-specific E3 ubiquitin ligases strongly induced during muscle atrophy. Consistent with these findings, both *Fbxo32* and *Trim63* transcripts were significantly upregulated in SCI muscles (Fig. 2**d**). EMS treatment alone induced only a partial reduction in atrogene expression, whereas the EMS+BoNT/A group displayed values comparable to naive animals. Notably, *Trim63* expression was significantly reduced not only compared with SCI mice but also compared with EMS+saline-treated animals, supporting an additional beneficial effect associated with BoNT/A administration.

As previously reported, collagen deposition is a hallmark of disuse-associated muscle atrophy [13]. Sirius red staining revealed a marked accumulation of collagen in muscles from untreated SCI mice, which was attenuated by EMS treatment and further improved in the EMS+BoNT/A group (Fig. 2**e**). In agreement with these observations, collagen type I alpha 1 (*Col1a1*) mRNA expression remained elevated in EMS+saline mice, whereas EMS+BoNT/A treatment restored transcript levels toward the naive condition and significantly reduced them compared with both SCI and EMS+saline groups (Fig. 2**f**).

Finally, SCI induced a marked loss of muscle innervation, evidenced by the reduced colocalization of bungarotoxin (BTX) and synaptophysin (Syn) signals at neuromuscular junctions (Fig. 2**g**). While EMS partially preserved neuromuscular connectivity, the EMS+BoNT/A group showed values comparable to naive mice together with a significant improvement compared with SCI animals.

Taken together, our findings highlight the potential of EMS, especially when combined with BoNT/A, as a promising rehabilitation strategy to counteract muscle atrophy, limit fibrotic changes, and preserve neuromuscular connectivity following severe SCI.

### 3.3 Mechanisms of BoNT/A neuroprotection: mitigation of excitotoxicity, astocytosis, and reduction of neuroinflammation

As previously demonstrated [13], and here confirmed (**Additional file 1:** Fig. S5), BoNT/A has long-lasting enzymatic activity in the spinal cord acting on different cell types. We examined the presence of cleaved SNAP25 (cl-SNAP25) in neuron-specific nuclear protein (NeuN)⁺ neurons and GFAP⁺ astrocytes within perilesional (T7–T13) and epicentral regions (T9–T11) 60 d post-injury. The yellow colocalization signal confirms persistent toxin activity in both neuronal and astrocytic populations. The detection of cl-SNAP25 at thoracic levels, distant from the lumbar injection site, supports the occurrence of retrograde migration of the toxin along the neuroaxis.

**Fig. 3:**
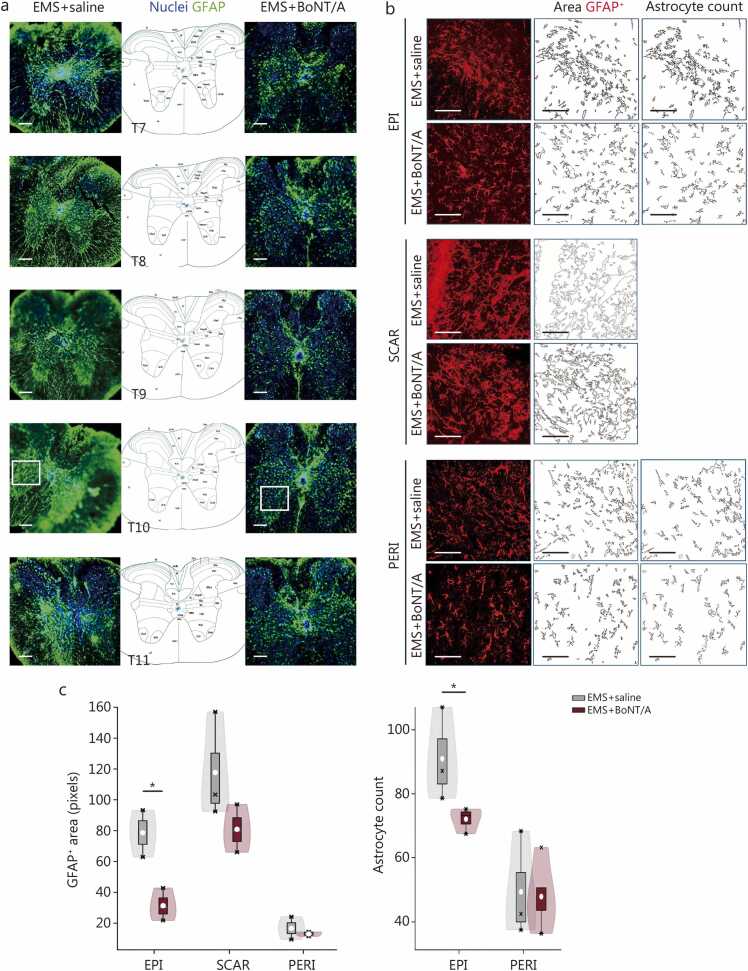
Modulation of astrocytic activation by BoNT/A at chronic stages after SCI. **a** Representative immunofluorescence images (10×, scale bar=100 μm) of GFAP^+^ astrocytes (green) and nuclei (blue) across thoracic spinal cord levels (T7–T11), 60 d after SCI. Animals underwent EMS starting at D3 post-injury and received either saline or BoNT/A at D15 post-lesion. GFAP signal appears diffusely increased in EMS+saline mice, particularly at the lesion epicenter (in this representative image at T10 but encompassing between T9–T11), perilesion regions were defined as spinal cord areas adjacent to, but not directly involved in, the lesion epicenter, extending rostrally to T7 and caudally to T13. Central columns show corresponding spinal cord atlas sections for anatomical reference. **b, c** Quantification of GFAP^+^ area and astrocyte counts. Scale bar=100 μm. GFAP images were converted to binary masks and processed using two segmentation thresholds to extract complementary structural information: a less restrictive threshold (“200 mask”) captured astrocyte somata together with GFAP^+^ proximal processes, providing the measurement of GFAP^+^ area (total GFAP^+^ pixels); a more restrictive threshold (“400 mask”) isolated astrocyte somata only, enabling automatic soma detection and astrocyte counting independent of processes or fragmented extensions. The masks shown next to the fluorescence panels illustrate these two segmentation outputs. A global comparison across treatment groups and regions (EPI, SCAR, PERI) using the Kruskal-Wallis test revealed a significant effect on GFAP^+^ area: *H*_5_=15.76, *P=*0.0076; Pairwise Mann-Whitney *U* tests showed: EPI: GFAP^+^ area significantly reduced in EMS*+*BoNT/A mice vs. EMS*+*saline (*P=*0.0495). Astrocyte number followed the same pattern, with a significant reduction in the epicenter of BoNT/A-treated animals (*P=*0.0495). ⁎*P<*0.05. EMS. Electrical muscle stimulation; BoNT/A. Botulinum neurotoxin type A; SCI. Spinal cord injury; GFAP. Glial fibrillary acidic protein; EPI. Epicenter; SCAR. Scar core region; PERI. Perilesional region.

To investigate whether BoNT/A modulates astrocytic responses after SCI, we examined GFAP immunoreactivity and astrocyte distribution across the EPI, the glial scar (SCAR), and PERI regions at thoracic levels T7–T11, 60 d post-lesion (Fig. 3**a, b**). Quantitative analysis revealed a region-specific effect of BoNT/A on astrocytic activation (Fig. 3**c**). In EMS+saline animals, GFAP staining showed the expected robust reactive profile at the lesion EPI, with dense GFAP^+^ processes and an elevated number of astrocytes. In contrast, EMS+BoNT/A treatment was associated with a marked reduction in both GFAP^+^ area and astrocyte counts specifically within the epicentral tissue. This attenuation was not as evident in the surrounding PERI areas, where GFAP coverage and astrocyte density remained comparable between groups, nor in the SCAR, where high structural heterogeneity limited detectability of treatment-related differences. Across EPI and PERI, the relationship between GFAP^+^ area and astrocyte number revealed a strong concordance (Spearman’s correlation analysis *ρ*=0.85, *P*<0.001), indicating that animals displaying more extensive GFAP immunoreactivity also showed higher astrocyte counts. Together, these results indicate that BoNT/A preferentially reduces astrocytic reactivity within the lesion EPI, where glial activation is typically most pronounced. Effects in perilesional and scar territories appear more modest or variable, likely reflecting the intrinsic heterogeneity and structural complexity of these regions following chronic SCI.

**Fig. 4:**
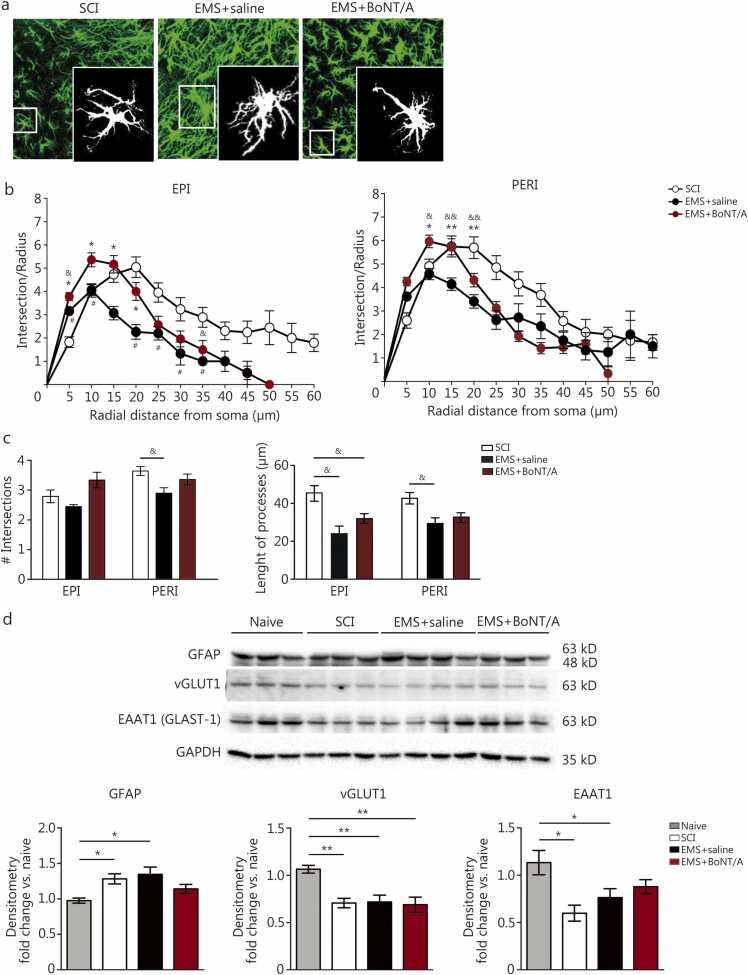
Astrocytic remodeling in the EPI and PERI regions following SCI and EMS-based treatments. **a** Representative extraction of GFAP^+^ cell from immunofluorescence images (40×, zoom 3×) and corresponding binary reconstructions illustrate astrocyte morphology. Sholl profiles were generated by quantifying GFAP^+^ intersections as a function of radial distance from the soma. **b** EPI: single-radius Mann-Whitney comparisons indicated significant differences between EMS+saline vs. EMS+BoNT/A at several proximal radii (5, 10, 15, 20 µm; *P<*0.001–0.0004). Relative to SCI animals, EMS-treated groups displayed altered astrocytic branching profiles, with EMS+BoNT/A differing at 5 and 35 µm (*P*<0.05), while EMS+saline differed at 5, 10, 20, 25, 30 and 35 µm (*P*<0.01). PERI: the Sholl analysis revealed significant differences restricted to the most proximal radii. A non-parametric Mann-Whitney test with FDR correction showed that SCI animals exhibited a significantly higher number of proximal intersections than both EMS+saline and EMS+BoNT/A groups at radii 0–5 µm. A marginal difference was also detected for SCI vs. EMS+saline at 20–25 µm, whereas no significant differences emerged between EMS+saline and EMS+BoNT/A at any radius after correction. ⁎*P<*0.05, ⁎⁎*P<*0.01, EMS+BoNT/A vs. EMS+saline; ^##^*P*<0.01, EMS+saline vs. SCI; ^&^*P*<0.05, ^& &^*P*<0.01, EMS+BoNT/A vs. SCI. Kruskal-Wallis tests confirmed group effects on both the number of intersections (*H*₅=11.14, *P=*0.0487) and the radial extent of processes (*H*₅=12.60, *P=*0.0274). Moreover, quantitative Sholl-derived metrics confirmed that epilesional astrocytes in SCI animals display a markedly expanded arbor, with significantly greater AUC, maximal radius and estimated process length compared to both EMS-treated groups (Kruskal-Wallis *P<*0.0001; Mann-Whitney SCI vs. EMS+saline *P*<0.0001; SCI vs. EMS+BoNT/A *P<*0.0001). **c** Summary bar graphs. Average intersections and the average of processes length (EPI and PERI) are shown for each group. Mann-Whitney post hoc tests revealed: ^&^*P<*0.05. *n*=3/4 group, slice/animal ≥5, cells/slice ≥3. All slice-level values were averaged per animal, which was considered the experimental unit). **d** Western blotting representative immunoblots for GFAP, vGLUT1, and EAAT1 (GLAST-1) with GAPDH loading control. Densitometric quantification (fold change vs. naive) showed: GFAP: ANOVA *F*_3,21=_4.705, *P=*0.0115; Tukey-Kramer: naive vs. SCI *P<*0.05, naive vs. EMS+saline *P<*0.05. vGLUT1: *F*_3,21=_7.68, *P=*0.0012; Tukey-Kramer: naive vs. SCI *P<*0.001, naive vs. EMS+saline *P<*0.001, naive vs. EMS+BoNT/A *P<*0.001. EAAT1: *F*_3,21_= 5.102, *P=*0.0083; naive vs. EMS+saline *P<*0.05, naive vs. SCI *P<*0.05 (naive, *n*=6; SCI, *n*=4; EMS+saline, *n*=8; EMS+BonT/A, *n*=5). ⁎*P<*0.05, ⁎⁎*P<*0.001. EMS. Electrical muscle stimulation; BoNT/A. Botulinum neurotoxin type A; SCI. Spinal cord injury; GFAP. Glial fibrillary acidic protein; EPI. Epicenter; PERI. Perilesional region; vGLUT1. Vesicular glutamate transporter 1; EAAT1/GLAST-1. Excitatory amino acid transporter 1; FDR. False discovery rate.

To further characterize astrocytic morphology, we performed Sholl analysis on GFAP^+^ cells from the different treatment groups (Fig. 4**a-c**). The analysis of GFAP^+^ astrocytes revealed a region- and treatment-dependent remodeling of astrocytic morphology. In the EPI area (Fig. 4**b**), all injured groups showed increased intersections close to the soma, but SCI animals displayed the most pronounced increase in proximal GFAP^+^ process intersections around the soma than both EMS-treated groups. Within the EMS conditions, astrocytes from EMS+BoNT/A mice tended to show a slightly higher proximal intersection/radius profile but with a more compact arbor, as suggested by the reduced radial extension and process length. This pattern indicates that BoNT/A does not abolish proximal branching but rather favors dense, short-range processes around the soma. A similar organization emerged in the PERI area (Fig. 4**b**). Here, SCI astrocytes again exhibited the largest and most extended arbors, whereas both EMS treatments reduced the radial spread of processes. Notably, EMS+BoNT/A astrocytes combined relatively high proximal intersections with a steeper decay of the Sholl curve and shorter maximum radius, resulting in the shortest and most compact arbors among the injured groups. The bar graphs summarizing intersection number and process length (Fig. 4**c**) are consistent with this interpretation, highlighting a BoNT/A-associated confinement of astrocytic territorial extension both in the perilesioned rim and in the lesion core.

To further support the morphological findings, Western blotting analysis was performed (Fig. 4**d**). GFAP levels were elevated in both SCI and EMS+saline animals compared to naive controls, indicating persistent astrocytic activation, whereas EMS+BoNT/A did not differ from baseline, suggesting attenuation of injury-associated GFAP upregulation. Similarly, vesicular glutamate transporter 1 (vGLUT1), although classically associated with excitatory terminals [31], is also expressed at the astrocyte-synapse interface [32], [33] and is increasingly recognized as a marker linked to glutamate handling and neuroinflammatory activity [33]. All injured groups displayed a reduction in vGLUT1 compared to naive tissue, with EMS+BoNT/A showing the most pronounced decrease, consistent with treatment-associated modulation of glutamatergic interfaces. Excitatory amino acid transporter 1 (EAAT1/GLAST-1) [33], [34], a key astrocytic glutamate transporter, was also reduced after injury, particularly in SCI and EMS+saline mice, whereas EMS+BoNT/A tended to preserve expression levels, pointing to a potential partial preservation of glutamate-buffering capacity. Second gel and ponceau staining are present in **Additional file 1:** Fig. S6.

To confirm astrocytic-glutamatergic interactions, GFAP-vGLUT1 colocalization was evaluated, showing alteration after SCI and partially restored by BoNT/A (**Additional file 1:** Fig. S7).

Taken together, these results indicate that BoNT/A modulates astrocytic reactivity, influencing structural complexity, glutamatergic interfaces, and associated molecular markers within the injured spinal cord.

**Fig. 5:**
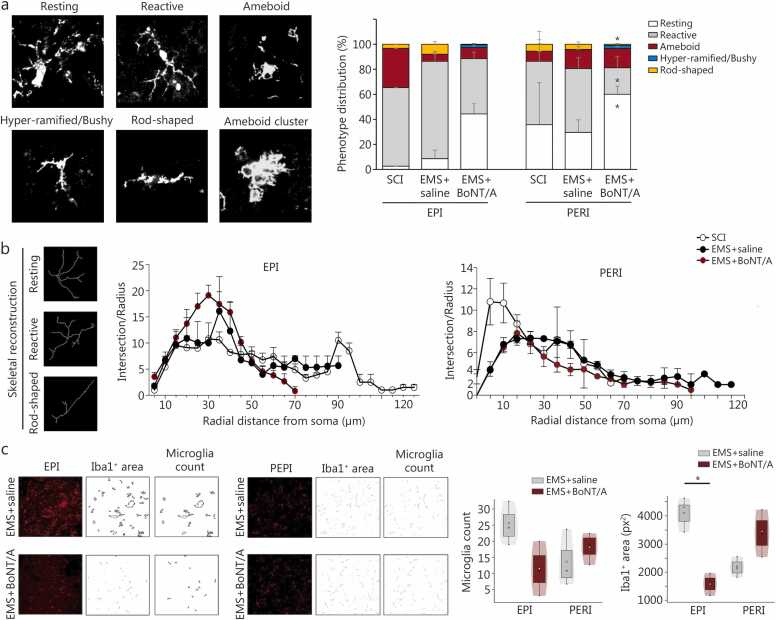
BoNT/A attenuates microglial activation and shifts microglial phenotype toward a resting state after SCI. **a** Microglial morphological phenotypes. Representative CD11b⁺/Iba1⁺ microglial morphologies identified in the EPI and PERI areas: resting/homeostatic, reactive, amoeboid (sometimes found as amoeboid clusters: aggregates of activated microglia with enlarged soma and retracted processes, characteristic of inflammatory/reactive states), hyper-ramified, and rod-shaped. Quantification (count for section-animal-level mean of multiple slices; experimental unit=animal divided for EPI and PERI) revealed significant treatment-dependent differences in phenotype distribution in the perilesioned region. Mann-Whitney *U* tests: Resting %: EMS+BoNT/A > EMS+saline *U*=0.000*, Z*=–1.964*, P*=0.0490*;* Reactive *%:* EMS+BoNT/A < EMS+saline; *U*=0.000, Z=–1.964, *P=*0.0495; Rod-shaped %: EMS+BoNT/A < EMS+saline *U*=0.000, Z=–1.964, *P=*0.0495. Other phenotypes showed no significant differences (SCI, *n*=2; EMS+saline, *n*=3, EMS+BoNT/A, *n*=3; ≥7 sections/treatment). **b** Representative skeletonized reconstructions of Iba1⁺ microglia, illustrating the marked morphological variability across groups and the features quantified in the subsequent Sholl analysis. Sholl analysis of microglia located within the lesion EPI. Sholl curves represent average intersections per radius (animal-level means across multiple slices; experimental unit=animal). As expected for microglia residing at the EPI, cells exhibited markedly higher branching densities and broader arborization profiles than in the perilesioned area). Global descriptors of arbor complexity (quantified as the number of intersections between microglial processes and concentric circles at increasing radial distances from the soma) computed on animal means did not reveal significant group differences: area under the curve (AUC, total intersections): Kruskal-Wallis *H*_2_=0.69, *P=*0.7070; Peak intersections: *H*_2_=1.00, *P=*0.6070; Peak radius (distance of maximal branching from soma): *H*_2_=2.88, *P=*0.2370. However, when the analysis was restricted to the 20–40 µm radial range, encompassing the main branch-density peak, a significant overall group effect emerged (radius-averaged intersections: Kruskal-Wallis *H*_2_=10.98, *P*=0.0041), indicating a general tendency of EMS+BoNT/A microglia to display increased branching complexity within this proximal domain compared with the other groups. Although post-hoc analyses at individual radial distances did not reach statistical significance, EMS+BoNT/A-treated microglia consistently exhibited the highest intersection counts across the 20–40 µm interval, SCI microglia the lowest, and EMS+saline an intermediate profile, collectively supporting a BoNT/A-associated enhancement of arbor complexity at the lesion EPI.*.* Sholl analysis of perilesioned microglia. Sholl curves reflect average intersections per radius (animal-level mean of multiple slices; experimental unit=animal). No significant group differences emerged in global arbor complexity: AUC (total intersections): Kruskal-Wallis *H*_2_=5.14, *P=*0.0766; Peak intersections: *H*_2_=4.03, *df*=2, *P=*0.1335. However, Peak radius (distance of maximal branching from soma) differed significantly among groups. Peak radius: *H*_2_=6.25, *P=*0.0439. Post-hoc interpretation: SCI microglia reached their maximum branching closer to the soma (more compact, proximally hypertrophic profile), while both EMS-treated groups exhibited more distal branching. EMS+BoNT/A presented an intermediate profile between SCI and EMS+saline (SCI, *n*=2; EMS+saline, *n*=3; EMS+BoNT/A, *n*=3; EPI≥7, PERI≥2, sections/treatment). **c** Microglial counts and Iba1⁺ area. Box/violin plots show microglial density and Iba1⁺ area in EPI and PERI. For cell counts no significant differences were detected. For Iba1⁺ area EPI region: BoNT/A showed a significant reduction in microglial area compared with saline *U*=0.000, *Z*=–1.964, *P=*0.0495 (SCI, *n*=2; EMS+saline, *n*=3; EMS+BoNT/A, *n*=3; ≥7 sections/treatment). For PERI region, no significant difference, although BoNT/A tended to show larger areas. The larger Iba1⁺ area observed in the perilesioned EMS+BoNT/A group is compatible with its greater proportion of ramified microglia, which possess more extended arborisation. Conversely, saline tissue displayed more rod-shaped, reactive, and amoeboid morphologies, smaller, compact cell types that reduce the measured Iba1⁺ area despite being more activated. ⁎*P<*0.05. EMS. Electrical muscle stimulation; BoNT/A. Botulinum neurotoxin type A; SCI. Spinal cord injury; EPI. Epicenter; PERI. Perilesional region; Iba1. Ionized calcium binding adaptor moleclule 1.

Using high-resolution Iba1/CD11b imaging, microglial cells were categorized into 5 morphological phenotypes [35] (Fig. 5**a**). 1) Resting (homeostatic): small soma with long, thin, highly branched processes that actively survey the surrounding environment; 2) Reactive: thicker and fewer processes, with a mildly enlarged soma, typical of inflammatory activation; 3) Amoeboid: rounded cells with minimal processes, associated with phagocytosis and strong immune activation; 4) Hyper-ramified: numerous long and thin processes, a morphology often linked to stress-related or transitional states; and 5) Rod-shaped: elongated, polarized cells with very few processes, frequently aligned in chains and associated with chronic neuroinflammation or neurodegenerative conditions. Across chronic SCI tissue, multiple phenotypes coexisted within both epilesioned and perilesioned regions, though with different distributions (Fig. 5**a**). In the EPI, EMS+saline animals also showed an enrichment of rod-shaped cells, while EMS+BoNT/A maintained a larger proportion of ramified morphologies (trend, *P*=0.08). In the PERI, EMS+BoNT/A treatment produced a redistribution of morphology-based classifications toward a higher proportion of resting/homeostatic profiles, whereas EMS+saline-treated animals exhibited a higher prevalence of reactive, ameboid, and rod-shaped microglia.

Sholl profile quantification on microglia located in proximity to the contusion epicenter (EPI, T9–T11) (Fig. 5**b**) revealed a distinct pattern of branching complexity (quantified as the number of intersections between microglial processes and concentric circles at increasing radial distances from the soma) compared with the perilesioned region. At the EPI, microglia from EMS+BoNT/A-treated mice displayed a broader and more distal branching profile, with increased process expansion within the proximal radial domain, whereas SCI microglia exhibited the most compact and centrally collapsed morphology. EMS+saline microglia showed an intermediate pattern, indicating partial rescue of arbor structure following EMS alone. Overall, these profiles are consistent with a continuum ranging from the highly hypertrophic, process-retracted morphology typical of EPI SCI microglia to a more extended, surveillance-like architecture restored by EMS+BoNT/A treatment.

Sholl profile quantification of microglia within the perilesional region, corresponding to areas not directly involved in the contusion (PERI, T7–T13) (Fig. 5**b**) did not reveal statistically significant differences between groups. However, the overall pattern indicated that EMS+saline-treated microglia displayed a more extended intersection/radius profile compared with BoNT/A, reflecting more compact arborisation in the toxin-treated group. This aligns with the coexistence of multiple phenotypes in chronic SCI tissue; hyper-ramified, reactive, and rod-like morphologies contribute to heterogeneous branching patterns that increase variability and dampen group-level statistical contrast.

Microglial cell counts (Fig. 5**c**) did not differ significantly between groups in either region. In contrast, the Iba1^+^ area was significantly reduced in the EPI region following EMS+BoNT/A treatment, indicating a lower burden of activated/reactive microglia compared with saline. In the PERI region, BoNT/A showed a trend toward increased Iba1^+^ area, consistent with the higher prevalence of ramified cells, which typically exhibit broader arborisation and therefore occupy a larger two-dimensional area despite being less reactive.

Importantly, EMS+saline-treated tissue showed multiple clusters of amoeboid and rod-shaped microglia, smaller and more compact reactive phenotypes that likely contribute to the reduced area measurements and to the higher variability observed in morphometric outcomes (area and branching) in chronic SCI, whereas the overall pattern emerging from morphology, area quantification, and Sholl profiles indicates that BoNT/A treatment is associated with a modulation toward less reactive, homeostatic-like ramified microglia, particularly in the perilesioned region, in contrast to the predominance of reactive amoeboid and rod-like phenotypes observed in saline controls.

For an integrated overview, the main results of EMS+BoNT/A on glial modulation are summarized in Table 2.

**Table 2:** Summary of region-specific glial modulation induced by combined EMS+BoNT/A treatment in chronic SCI.

**Compartment**	**Parameter**	**Comparison**	**Result**	**Direction**	**Interpretation**
EPI-restricted astroglial modulation					
EPI	GFAP^+^ area	EMS+BoNT/A vs. EMS+saline	Significant	↓ In BoNT/A	Reduced astroglial reactivity at EPI
	Astrocyte count	EMS+BoNT/A vs. EMS+saline	Significant	↓ In BoNT/A	Decreased astrocyte density at EPI
SCAR	GFAP^+^ area	EMS+BoNT/A vs. EMS+saline	Not significant	=	No detectable modulation in scar region
PERI	GFAP^+^ area	EMS+BoNT/A vs. EMS+saline	Not significant	=	No detectable modulation in perilesion
EPI+PERI	GFAP area vs. cell number correlation	All groups	Strong correlation	Positive	GFAP area reflects astrocyte density
Partial modulation of reactive astrocytic morphology					
EPI	Intersections (proximal radii)	EMS+saline vs. EMS+BoNT/A	Significant	Different	Morphological modulation by BoNT/A
	AUC (Sholl)	SCI vs. EMS+saline	Significant	↑ In SCI	Increased arbor complexity after SCI
	Process length	SCI vs. EMS+saline	Significant	↑ In SCI	Elongated processes in SCI
PERI	Proximal intersections (0–5 µm)	SCI vs. EMS+saline groups	Significant	↑ In SCI	Increased proximal branching after SCI
Western blotting spinal cord	GFAP	Naive vs. SCI; Naive vs. EMS+saline	Significant	↑ After SCI	Global increase of GFAP after injury
	vGLUT1	All injured vs. naive	Significant	↓ After SCI	Reduced excitatory synaptic marker after injury
	EAAT1/GLAST	SCI & EMS+saline vs. Naive	Significant	↓ After SCI	Reduced glutamate transporter expression after injury
Homeostatic-like microglia rebalancing					
EPI	Iba1^+^ area	BoNT/A vs. EMS+saline	Significant	↓ In BoNT/A	Reduced microglial activation signal
	Sholl (20–40 µm)	All groups	Significant	Different	Morphological differences among groups
PERI	Resting microglia %	BoNT/A vs. EMS+saline	Significant	↑ In BoNT/A	Increased resting-like microglial proportion
	Reactive microglia %	BoNT/A vs. EMS+saline	Significant	↓ In BoNT/A	Reduced reactive microglial phenotype
	Rod-shaped %	BoNT/A vs. EMS+saline	Significant	↓ In BoNT/A	Reduced rod-shaped morphology
	Peak radius	All groups	Significant	Different	Altered branching distribution

“=” indicates no change; “↑” indicates increased expression; “↓” indicates reduced expression. The table summarizes the principal glial endpoints (Fig. 3, Fig. 4, Fig. 5**)** significantly affected by the combined EMS+BoNT/A treatment. The table reports compartment-specific analyses (EPI, PERI, SCAR), the statistical comparisons performed, and the direction of change. Directional arrows indicate variation relative to the specified comparator. These summary interpretations reflect the dominant trend emerging from each dataset and should not be construed as evidence of complete or irreversible cell-state transitions. AUC. Area under the curve; BoNT/A. Botulinum neurotoxin type A; EAAT1. Excitatory amino acid transporter 1; EMS. Electrical muscle stimulation; GFAP. Glial fibrillary acidic protein; GLAST. Glutamate aspartate transporter (see EAAT1); Iba1. Ionized calcium binding adaptor moleclule 1; SCI. Spinal cord injury; vGLUT1. Vesicular glutamate transporter 1; EPI. Epicenter; SCAR. Scar core region; PERI. Perilesional region

### 3.4 BoNT/A reduces apoptotic cell death and promotes oligodendrocyte survival and differentiation after SCI

**Fig. 6:**
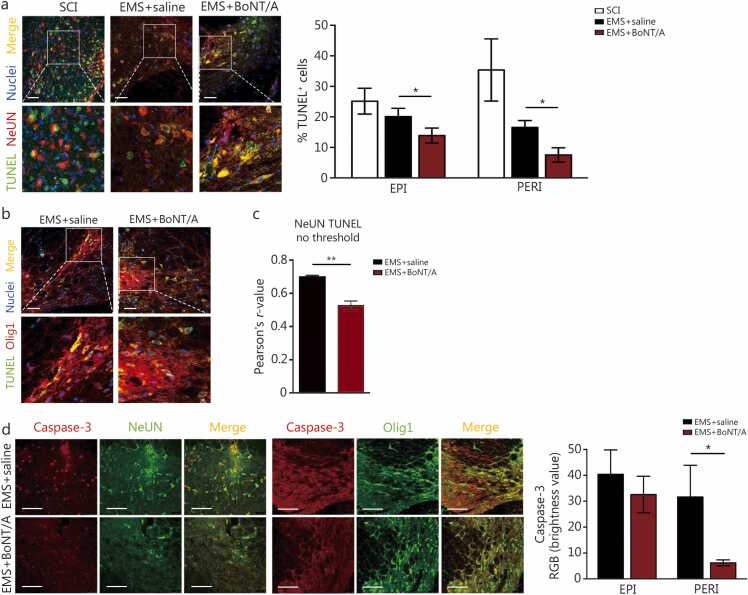
BoNT/A treatment reduces apoptotic cell death and preserves tissue integrity following SCI. **a, b** Representative immunofluorescence images of transverse spinal cord sections stained for TUNEL (green), DAPI (blue), NeuN or Olig1 (red) and merged channels from SCI, EMS+saline, and EMS+BoNT/A groups. Scale bar=50 mm. Quantitative analysis of apoptosis based on TUNEL staining. The percentage of apoptotic cells was calculated from the automated counts of TUNEL⁺ cells and total nuclei acquired for each slice. TUNEL staining was quantified in the EPI (T9–T11) and in PERI (T7–T8 and T12–T13) in SCI, EMS+saline, and EMS+BoNT/A mice. Kruskal-Wallis analysis revealed a significant overall difference among groups (*H*₅=12.47, *P=*0.0289), and post hoc pairwise comparisons (Mann-Whitney test) showed a significant reduction in apoptotic cells in EMS+BoNT/A compared with EMS+saline in both EPI and PERI regions (*P<*0.05). **c** Quantification of TUNEL-NeuN co-localization analysis. Graphs show a significant reduction in co-localization between neuronal marker NeuN and apoptotic TUNEL signal in BoNT/A-treated animals, assessed via Pearson’s correlation (no threshold, unpaired Student’s *t*-test: *t*_19_=2.882, *P*<0.001). **d** Representative images (40×) of caspase-3 (red) immunoreactivity and its co-localization with NeuN or Olig1 (green). Scale bar=100 μm. RGB (red, green, blue) intensity analysis did not show an overall significant difference among groups (Kruskal-Wallis test). However, post hoc pairwise comparisons revealed a significant reduction in signal intensity in the perilesional spinal regions of EMS+BoNT/A-treated animals compared with EMS+saline mice (*P*<0.05). SCI: *n*=2, EMS+saline and EMS+BoNT/A: *n*=3–4, with a minimum of 10 images analyzed for each animal, all slice-level values were averaged per animal, which was considered the experimental unit. ⁎*P*<0.05, ⁎⁎*P*<0.001. EMS. Electrical muscle stimulation; BoNT/A. Botulinum neurotoxin type A; SCI. Spinal cord injury; EPI. Epicenter; PERI. Perilesional region; DAPI. 4’,6-diamidino-2-phenylindole; NeuN. Neuron-specific nuclear protein; Olig1. Oligodendrocyte lineage transcription factor 1; TUNEL. Terminal deoxynucleotidyl transferase dUTP nick end labeling.

To assess whether astrocyte remodelling, glutamatergic modulation, and neuroinflammation reduction by BoNT/A contribute to neuroprotection, we evaluated apoptotic cell death in spinal cord tissue 60 d post-injury using TUNEL staining. Immunofluorescence analysis revealed a high density of TUNEL^+^ nuclei (green) in both the SCI and EMS+saline groups, while markedly fewer apoptotic cells were observed in the EMS+BoNT/A group (Fig. 6**a**). The spared parenchyma appeared more compact and better organized in BoNT/A-treated animals. Quantification confirmed a general reduction in the number of apoptotic nuclei [TUNEL⁺/4′,6-diamidino-2-phenylindole (DAPI)] in the EMS+BoNT/A group compared to both SCI and EMS+saline groups (Fig. 6**a**). This decrease was significant not only at the lesion EPI (T9–T11) but also in more distant PERI (T12–T13 and T7–T8). These findings support a pro-survival and neuroprotective action of BoNT/A within the injured spinal cord. To better illustrate how apoptotic cells distribute relative to the total number of nuclei across the tissue, individual values were plotted separately for the dorsal horn (DH) and ventral horn (VH), two regions that differ markedly in cellular density and composition, in the slice-wide distribution graph shown in **Additional file 1:** Fig. S8a.

To determine whether this effect reflects preferential protection of specific cell types, we assessed the degree of co-localization between neuronal nuclei (NeuN⁺) and TUNEL staining. Pearson’s correlation analyses revealed significantly reduced co-localization in the EMS+BoNT/A group compared to EMS+saline, suggesting that BoNT/A may preferentially protect neurons from apoptosis (Fig. 6**b, c**). Additional analyses using thresholded Manders’ coefficients showed consistent trends but did not reach statistical significance (**Additional file 1:** Fig. S8b).

We next examined the expression of the executioner protease caspase-3 in the spinal cord (Fig. 6**d**) parenchyma. Immunofluorescence analysis didn’t reveal a qualitative reduction of caspase-3 signal for all analysed sections. However, a significant reduction in caspase-3 signal intensity in the PERI regions of EMS+BoNT/A-treated animals compared with EMS+saline mice was appreciable. These findings indicate that BoNT/A reduces caspase-related apoptotic signaling specifically in areas surrounding the lesion, consistent with its broader neuroprotective profile.

**Fig. 7:**
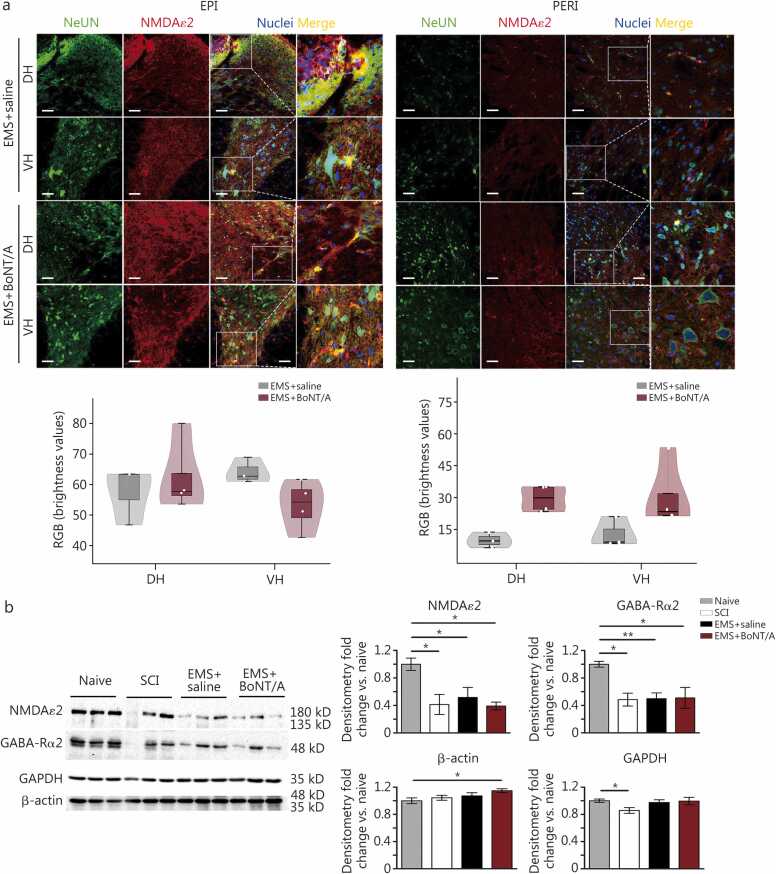
Expression of NMDA and GABA-A receptor subunits in the spinal cord 60 d after SCI. **a** Representative confocal images showing NMDAε2 immunoreactivity in the dorsal (DH) and ventral horns (VH) of EPI (T9–T11) and PERI (T7–T13) spinal cord regions. Sections were co-stained for NeuN (green), NMDAε2 (red), and nuclei (DAPI, blue). EPI corresponds to the region directly affected by the trauma, whereas PERI includes adjacent rostral-caudal spinal segments within 2–3 mm from the impact zone. Scale bar=50 µm. Quantitative analysis of NMDAε2 fluorescence intensity in DH and VH across EPI and PERI. Each point represents one animal (*n*=3–4). All slice-level values (two per animal for each area and two DH/VH measurements per slice) were averaged per animal, which was considered the experimental unit. Box plots display median (IQR), and violin plots illustrate data distribution. Kruskal-Wallis analysis indicated a significant overall difference among the four groups for both DH (*P*=0.0117) and VH (*P*=0.0139); however, no pairwise comparison remained significant after Holm correction. Scheirer-Ray-Hare testing revealed a main effect of area (EPI vs. PERI) on both DH (*P*=0.0017) and VH (*P*=0.0060), with no significant main effect of treatment and no significant interaction (trend for VH, *P*=0.080). **b** Western blotting analysis (*n=*6–7) of excitatory (NMDAε2) and inhibitory (GABA-Rα2) receptor subunits in spinal cord lysates collected 60 d after injury. Representative blots show protein levels across groups. Quantification was performed on all available samples (naive, *n=*6; SCI, *n=*4; EMS+saline *n=*7; EMS+BoNT/A, *n=*4) and expressed as fold-change relative to the naive mean. Both receptors were significantly modulated in injured groups vs. naive controls, with no major differences among SCI, EMS+saline, and EMS+BoNT/A SCI mice (one-way ANOVA: NMDAε2 *F*_3,21=_5.48, *P=*0.0061; GABA-Rα2 *F*_3,21=_2.34, *P=*0.0037). β-actin and GAPDHlevels also varied across groups (unpaired *t*-tests: β-actin EMS+BoNT/A vs. naive *t*₁₀=2.91, *P=*0.0061; GAPDH SCI vs. naive *t*₁₀=2.92, *P=*0.0154). Protein loading was verified by Ponceau staining (see **Additional file 1:**Fig. S5). ⁎*P*<0.05; ⁎⁎*P*<0.001. EMS. Electrical muscle stimulation; BoNT/A. Botulinum neurotoxin type A; SCI. Spinal cord injury; NMDA: N-methyl-D-aspartate; GABA-A: Gamma-aminobutyric acid type A receptor; EPI. Epicentre; PERI. Perilesional region; IQR. Interquartile range; DAPI. 4’,6-diamidino-2-phenylindole.

To investigate whether the observed neuroprotection could involve restoration of excitatory/inhibitory receptor balance, we analyzed the expression and distribution of the N-methyl-D-aspartate (NMDA)ε2 and the GABA-Rα2 in the spinal cord 60 d after injury (Fig. 7**a, b**). Immunofluorescence analysis of NMDA receptor (NMDAε2) distribution (Fig. 7**a**) revealed the spatial organization of NMDAε2 labeling within the dorsal (DH) and ventral (VH) horns of both EPI and PERI. Representative images and RGB brightness quantification revealed regional differences in NMDAε2 immunoreactivity between EPI and PERI areas in both EMS+saline and EMS+BoNT/A groups. Although post hoc analysis did not identify statistically significant differences between treatments, PERI sections from EMS+BoNT/A mice showed numerically higher RGB values. A similar regional distribution pattern was observed for GABA-Rα2 immunoreactivity, although no evident differences between EMS+saline and EMS+BoNT/A groups were detected. (**Additional file 1:** Fig. S9). Western blotting analysis (Fig. 7**b**) confirmed that both NMDAε2 and GABA-Rα2 receptor subunits were significantly downregulated in all injured groups compared to naive controls, with no differences among SCI, EMS+saline, and EMS+BoNT/A animals, thereby confirming a persistent synaptic imbalance that is not corrected by BoNT/A treatment. Interestingly, we also observed treatment- and injury-dependent modulation of β-actin and glyceraldehyde-3-phosphate dehydrogenase (GAPDH), two commonly used housekeeping proteins. Specifically, GAPDH levels were reduced in SCI tissue, while β-actin was increased in the EMS+BoNT/A group (**Additional file 1:** Fig. S9). These changes may reflect broader cytoskeletal and metabolic remodelling processes in response to injury and treatment. Notably, normalization of receptor signals to GAPDH abolished statistical significance (data not shown), highlighting the potential instability of conventional loading controls in lesioned tissue. Ponceau staining confirmed consistent protein loading across lanes (**Additional file 1:** Fig. S10).

**Fig. 8:**
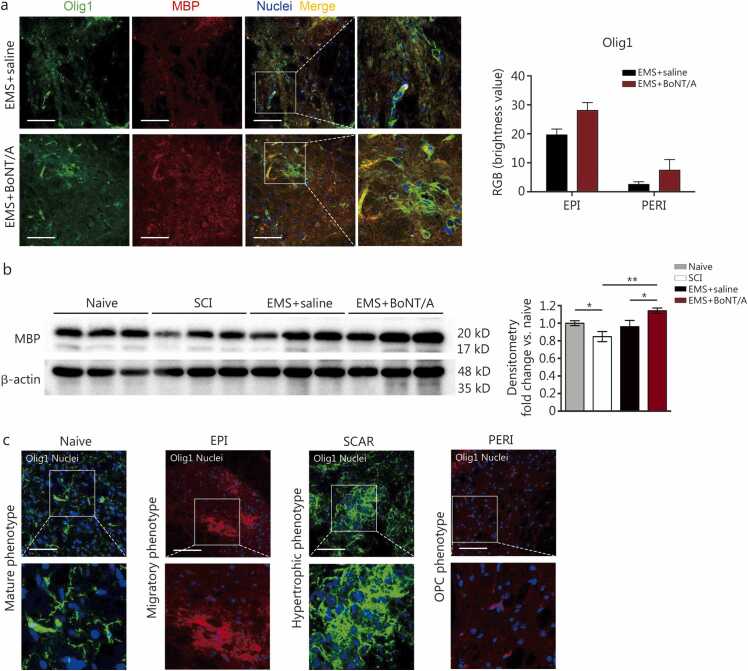
BoNT/A modulates Olig1 expression and enhances myelin protein expression in the chronic phase of SCI. **a** Representative immunofluorescence images (scale bar=100 mm) of spinal cord sections stained for Olig1 (green), MBP (red), and DAPI (blue, nuclei) from EMS+saline and EMS+BoNT/A groups, acquired 60 d after injury. Merged images and 3× zoom-in panels illustrate Olig1⁺ cells and enhanced MBP signal in BoNT/A-treated animals. Quantification of fluorescence brightness values, separately evaluated in the EPI or PERI shows a trend, although not significant, in increased Olig1 expression in the EMS+BoNT/A group compared to EMS+saline. *n=*2/3 animals/treatment, Saline: 12–40 slices, BoNT/A: 10–36 slices; all slice-level values were averaged per animal, which was considered the experimental unit). **b** Representative Western blotting of MBP in spinal cord lysates from all experimental groups (*n=*3 per group shown in blot). Densitometric analysis of MBP levels (normalized to the average of naive controls) is shown in the bar graph, including all samples: naive (*n=*6), SCI (*n=*4), EMS+saline (*n=*7), EMS+BoNT/A (*n=*4). One-way ANOVA revealed a significant group effect (*F*_3,21=_5.153; *P=*0.0079), with Tukey-Kramer post hoc test indicating a significant increase of MBP in the EMS+BoNT/A group compared to SCI (*P<*0.01). The modulation of MBP expression is not statistically significant when normalized to β-actin, due to variability in housekeeping gene levels across groups (as shown in Fig. 7). Protein loading was assessed via Ponceau staining (**Additional file 1:**Fig. S10), and a replicate Western blotting is shown in **Additional file 1:**Fig. S10. **c** Distinct Olig1-expressing oligodendrocyte phenotypes were similarly observed in both EMS+saline and EMS+BoNT/A mice. For this reason, the representative image was selected randomly, as both groups displayed comparable phenotypic features. Confocal images (40×, scale bar=100 μm) illustrate Olig1 staining in the EPI- and PERI-lesion areas, as well as in SCAR and naive tissue, where changes in the oligodendrocyte phenotypic profile are appreciable. The RGB (red, green, blue) values shown in panel **a** reflect phenotype-specific changes in oligodendrocytes driven by the injury and by the anatomical region affected. For this reason, areas corresponding to the glial scar were excluded from the RGB-based analysis. ⁎*P*<0.05, ⁎⁎*P*<0.001. EMS. Electrical muscle stimulation; BoNT/A. Botulinum neurotoxin type A; SCI. Spinal cord injury; EPI. Epicentre; PERI. Perilesional region; DAPI. 4’,6-diamidino-2-phenylindole; Olig1. Oligodendrocyte lineage transcription factor 1; MBP. Myelin basic protein;.

To further characterize the cytoprotective effects of BoNT/A, we examined oligodendroglial survival and myelin protein expression in the injured spinal cord 60 d post-injury. Immunofluorescence for oligodendrocyte lineage transcription factor 1 (Olig1) revealed (Fig. 8**a**) a higher density and intensity of Olig1⁺ cells in EMS+BoNT/A-treated animals compared to EMS+saline and the evaluation of fluorescence brightness of Olig1 expression in the BoNT/A group reveal an increasing trend, although not significant both in EPI or PERI areas.

To determine whether BoNT/A also influenced oligodendrocyte maturation and myelin production, we analyzed myelin basic protein (MBP) expression. Western blotting results showed a significant increase in MBP levels in EMS+BoNT/A animals compared to all other groups, including naive, SCI, and EMS+saline (Fig. 8**b**). Quantitative densitometry confirmed this effect, indicating enhanced myelin protein synthesis in response to BoNT/A treatment. Of interest, morphological observation (Fig. 8**c**) of spinal cord sections collected from proximal (EPI) and distal (PERI) regions, as well as from the glial scar, in both EMS+saline- and EMS+BoNT/A-treated mice revealed a marked shift in Olig1 localization and oligodendrocytes phenotype, consistent with previous reports [36], [37], [38].

**Fig. 9:**
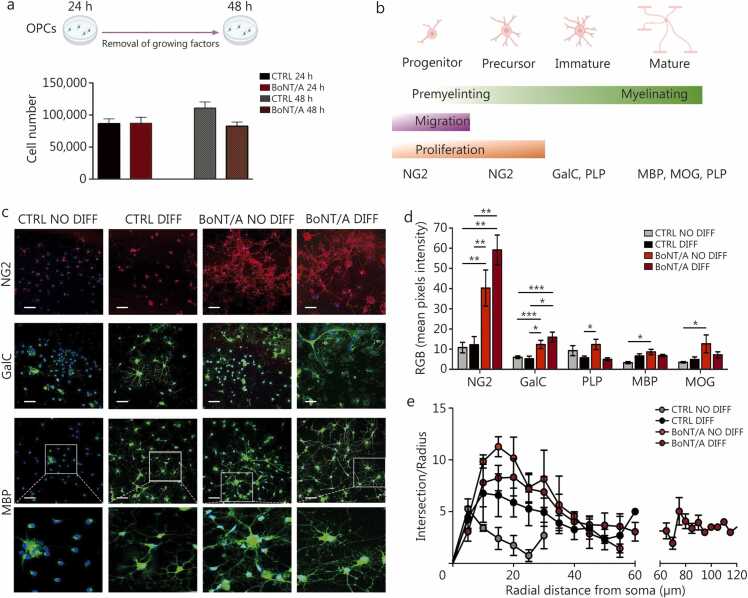
BoNT/A enhances oligodendroglia differentiation and morphological complexity *in vitro*. **a** Timeline of the experimental protocol: oligodendrocyte precursor cells (OPCs) were cultured under proliferative conditions for 24 h and then switched to differentiating conditions (48 h), in the presence or absence of BoNT/A. **b** Schematic of the oligodendrocyte lineage progression: OPCs differentiate into pre-oligodendrocytes, immature oligodendrocytes, and finally mature myelinating cells. Marker expression during this process includes neuron-glial antigen 2 (NG2, OPC marker), galactocerebroside C (GalC, immature OL marker), proteolipid protein (PLP), myelin basic protein (MBP), and myelin oligodendrocyte glycoprotein (MOG) as markers of mature myelinating oligodendrocytes. **c** Representative confocal images (40×, scale bar=50 μm) of NG2 (red), GalC (green), and MBP (green) staining in control and BoNT/A-treated cultures under differentiating (DIFF) and non-differentiating (NO DIFF) conditions. Nuclei are counterstained with DAPI (blue). High magnification images (zoom 3×) of MBP immunofluorescence (showed in area in the square) highlighting the increased complexity and arborization in BoNT/A-treated cells. **d** Quantitative analysis of immunofluorescence intensity (mean RGB values: *n=*3 group; 6–12 images evaluation/group) revealed the following: NG2: ANOVA *F*_3,20_=13.834, *P<*0.0001. Tukey-Kramer post-hoc: BoNT/A NO DIFF vs. CTRL NO DIFF *P<*0.005; BoNT/A NO DIFF vs. CTRL DIFF *P<*0.005; BoNT/A DIFF vs. CTRL NO DIFF *P<*0.0001; BoNT/A DIFF vs. CTRL DIFF *P<*0.0001. GalC: ANOVA *F*_3,34_=8.037, *P=*0.0004. Tukey-Kramer post-hoc BoNT/A NO DIFF vs. CTRL NO DIFF *P<*0.001; BoNT/A NO DIFF vs. CTRL DIFF *P*<0.05; BoNT/A DIFF vs. CTRL NO DIFF *P*<0.0005; BoNT/A DIFF vs. CTRL DIFF *P*<0.05. PLP: ANOVA *F*_3,16=_4.089, *P=*0.0248. Tukey-Kramer post-hoc BoNT/A NO DIFF vs. CTRL DIFF *P*<0.05. MBP: ANOVA *F*_3,21_= 4.145, *P=*0.0187. Tukey-Kramer post-hoc BoNT/A NO DIFF vs. CTRL NO DIFF *P*<0.05. MOG: ANOVA *F*_3,20_=2.59, *P=*0.081. Tukey-Kramer post-hoc BoNT/A NO DIFF vs. CTRL NO DIFF *P*<0.05. **e** Sholl morphometric analysis of MBP⁺ cells performed using the neuroanatomy plugin in FIJI/ImageJ. Repeated measure ANOVA shows main effect for treatment (*F*_3,193_=98.53, *P*<0.0001), for radial distance (*F*_12,192_=60.08, *P*<0.0001), and interaction treatment × radial distance (*F*_36,192_=5.53, *P*<0.0001). Tukey-Kramer post-hoc test evidenced significant differences in number of intersections per radius: BoNT/A DIFF cells showed significantly increased arborization compared to CTRL NO DIFF and CTRL DIFF up to 20 µm from the soma (*P*<0.001). BoNT/A NO DIFF vs. CTRL NO DIFF at the same radial distance (*P*<0.0001) and for total process length: both BoNT/A DIFF and BoNT/A NO DIFF groups showed significantly longer total process length compared to CTRL NO DIFF (*P*<0.0001). ⁎*P<*0.05, ⁎⁎*P<*0.01, ⁎⁎⁎*P<*0.001. RGB. Red, green, blue; NO DIFF. Non-differentiating (proliferative) condition; DIFF. Cell differentiation.

To verify whether BoNT/A exerts a direct effect on the oligodendroglia lineage (Fig. 9), we exposed cultured OPCs to 10 pmol/L BoNT/A under either proliferative (NO DIFF) or differentiating (DIFF) conditions. Cell counts at 24- and 48-hours post-treatment showed no significant differences in OPC survival across groups (Fig. 9**a**), indicating that BoNT/A does not compromise cell viability.

Morphological analysis using immunofluorescence revealed distinct changes in marker expression across the maturation stages of the oligodendroglia lineage (Fig. 9**b, c**). Under control differentiating conditions, a partial increase in galactocerebroside C (GalC)⁺ and MBP⁺ cells was observed, consistent with normal progression toward immature and mature oligodendrocytes. However, BoNT/A treatment, both in proliferative and differentiating conditions, led to a significant upregulation of GalC, MBP, and myelin oligodendrocyte glycoprotein (MOG) immunoreactivity, suggesting accelerated differentiation and myelin acquisition. RGB (red, green, blue) quantification confirmed these effects (Fig. 9**d**). Expression evaluation confirmed that BoNT/A promotes the differentiation of oligodendrocyte lineage cells. In particular, GalC, a marker of immature oligodendrocytes, was strongly upregulated in both BoNT/A-treated groups, under proliferative (NO DIFF) and differentiating (DIFF) conditions, indicating a robust shift toward a premyelinating phenotype. Expression of MBP was also elevated following BoNT/A treatment, even in the absence of differentiation stimuli, suggesting an early initiation of myelin-related programs (Fig. 9**d**). MOG levels showed a similar trend, further supporting the progression toward a mature, myelin-producing oligodendrocyte phenotype. Notably, proteolipid protein (PLP), another marker of mature oligodendrocytes, was selectively increased in BoNT/A-treated cells maintained in proliferative conditions, reinforcing the ability of BoNT/A to drive maturation independently of extrinsic differentiation cues (representative images of PLP- and MOG-positive OPC staining are provided in **Additional file 1:** Fig. S11). In contrast, neuron-glial antigen 2 (NG2), a marker of OPCs, was significantly downregulated in all BoNT/A-treated groups, consistent with the exit from the progenitor state and commitment to the differentiation pathway.

To assess whether BoNT/A enhances morphological complexity, we performed Sholl analysis on MBP⁺ cells (Fig. 9**e**). BoNT/A-treated cells exhibited a significantly higher number of intersections at distances up to 20 μm from the soma compared to CTRL NO DIFF and CTRL DIFF. In addition, both BoNT/A NO DIFF and DIFF groups showed significantly increased total process length vs. CTRL NO DIFF, further supporting the toxin’s effect on promoting arborization and maturation. High-magnification images (Fig. 9**c**) confirmed the increased complexity and branching of MBP⁺ cells in BoNT/A-treated cultures, even under non-differentiating conditions.

To confirm that BoNT/A is internalized and enzymatically active in oligodendrocytes, we performed triple immunofluorescence for cl-SNAP25, MOG or PLP, and DAPI (**Additional file 1:** Fig. S11). Confocal imaging revealed clear colocalization of cl-SNAP25 signal within MOG⁺ and PLP⁺ cells, indicating that BoNT/A enters oligodendroglia cells and cleaves SNAP25, its canonical substrate. This result provides direct evidence of BoNT/A internalization and activity in cells of the oligodendrocyte lineage.

Taken together, these *in vitro* findings demonstrate that BoNT/A directly promotes the differentiation of OPCs toward mature myelinating oligodendrocytes.

For an integrated overview, the main results of EMS+BoNT/A on neuroprotection are summarized in Table 3.

**Table 3:** Summary of neuroprotection and oligodendroglial responses induced by combined EMS+BoNT/A treatment in chronic SCI.

**Compartment**	**Parameter**	**Comparison**	**Result**	**Direction**	**Interpretation**
Reduction of apoptosis-associated markers					
EPI	TUNEL^+^ cells	EMS+BoNT/A vs. EMS+saline	Significant	↓ In BoNT/A	Reduced apoptotic signal
	NeuN+/TUNEL+ colocalization	EMS+BoNT/A vs. EMS+saline	Significant	↓ In BoNT/A	Reduced neuronal apoptotic involvement
	Olig1+ density	EMS+BoNT/A vs. EMS+saline	Significant	↑ In BoNT/A	Increased oligodendroglial presence
	Oligodendroglial maturation markers	EMS+BoNT/A vs. EMS+saline	Significant	↑ In BoNT/A	Enhanced maturation profile
PERI	TUNEL+ cells	EMS+BoNT/A vs. EMS+saline	Significant	↓ In BoNT/A	Reduced apoptotic signal
	Caspase-3 signal	EMS+BoNT/A vs. EMS+saline	Significant	↓ In BoNT/A	Reduced apoptotic marker expression
Pro-remyelinating oligodendroglial response					
PERI	Olig1+ density	EMS+BoNT/A vs. EMS+saline	Significant	↑ In BoNT/A	Increased oligodendroglial presence
Western blotting spinal cord	MBP	EMS+BoNT/A vs. EMS+saline	Significant⁎	↑ In BoNT/A	Increased myelin-associated protein
*In vitro* OPC differentiation	Differentiated phenotype	BoNT/A vs. control	Significant	↑ In BoNT/A	Promotion of oligodendroglial differentiation

⁎ Trend toward significance; “↑” indicate increased expression; “↓” indicate decreased expression. Table summarizes the principal apoptosis-related and oligodendroglial endpoints (Fig. 6, Fig. 8, Fig. 9) significantly modulated by EMS+BoNT/A treatment. The table reports the anatomical compartment analyzed, the statistical comparison performed, and the direction of change relative to the specified comparator. Descriptive summaries of the dominant treatment-associated trends are included where appropriate; these reflect the overall direction of statistically significant changes in apoptosis-associated markers and oligodendroglial maturation and do not imply direct quantification of net cell survival or complete remyelination. EMS. Electrical muscle stimulation; BoNT/A. Botulinum neurotoxin type A; TUNEL. Terminal deoxynucleotidyl transferase dUTP nick end labeling; NeuN. Neuron-specific nuclear protein; Olig1. Oligodendrocyte lineage transcription factor 1; MBP. Myelin basic protein; OPC. Oligodendrocyte precursor cell

## 4 Discussion

Chronic SCI remains one of the most challenging conditions in neurorehabilitation, as most patients live for decades with persistent neurological deficits, NeP, neuroinflammation, muscle atrophy, and systemic complications that severely impact quality of life [1], [2], [3]. According to the World Health Organization (WHO), an estimated 15.4 million people were living with SCI in 2021, highlighting the enormous long-term clinical and socioeconomic burden associated with the chronic phase of the disease [39]. Despite advances in acute SCI management, effective interventions targeting chronic SCI remain extremely limited. In this context, strategies capable of modulating glial dysfunction, chronic neuroinflammation, synaptic imbalance, and impaired plasticity represent a major translational need [6], [7].

Our work addresses this clinical gap by evaluating whether delayed intrathecal BoNT/A administration combined with EMS can reshape the chronic injury environment and restore cellular homeostasis within the injured spinal cord. This study provides preclinical evidence that the combination of early EMS and delayed BoNT/A treatment promotes substantial functional recovery in a severe model of chronic SCI.

In the SCI rehabilitation field, activity-based interventions such as locomotor training, including treadmill-based paradigms, aim to engage residual spinal circuitry through repetitive sensorimotor input [19], while neuromodulatory strategies such as epidural electrical stimulation attempt to reactivate spared spinal networks and restore voluntary locomotor control [40]. These approaches primarily rely on the preservation of residual functional connectivity.

In our severe contusion model, this approach did not translate into measurable recovery, as also indicated by our preliminary treadmill training experiments reported in the **Additional file 1: Methods** and Fig. S2, where BMS scores remained unchanged over the observation period. In this context, we deliberately prioritized EMS as a peripheral, muscle-directed strategy to address an early and major critical point of severe SCI, rapid disuse-driven atrophy and progressive loss of neuromuscular integrity, rather than attempting to directly “train” locomotion in paraplegic animals. By preserving muscle trophism and neuromuscular connectivity, EMS is expected to stabilize the effector system that ultimately executes movement, thereby increasing the likelihood that any centrally acting intervention can be functionally expressed. Animals receiving this combined approach (EMS+BoNT/A) showed significant restoration of hindlimb motor function, which was not observed in any other group, including those treated with EMS or BoNT/A alone. These findings underscore the importance of an integrative therapeutic strategy capable of both preserving tissue integrity and modulating the chronic neuroinflammatory environment.

Importantly, we demonstrate that a short-term EMS protocol, initiated early after injury, is sufficient to exert long-term protective effects against muscle atrophy, a key limiting factor in chronic SCI recovery. Although EMS does not induce persistent spinal or regenerative effects, its peripheral impact on the stimulated muscles is long-lasting, as evidenced by the sustained mitigation of atrophy and preservation of muscle structure observed even 60 d after stimulation cessation. Even when discontinued prior to BoNT/A administration, EMS alone preserved muscle structure and function, likely contributing to the success of subsequent interventions. This supports clinical observations that early EMS enhances tissue trophism, perfusion, and microenvironmental quality [41], [42] but goes further by showing that EMS creates a pro-regenerative niche that remains responsive to neuroactive therapies weeks later.

Beyond conventional rehabilitation [7], regenerative strategies aimed at modifying the chronic inhibitory milieu of SCI are increasingly explored. These include enzymatic degradation of inhibitory extracellular matrix components using chondroitinase ABC [43], as well as cell-based therapies designed to replace neuronal and glial populations or provide trophic support [44]. Such approaches attempt to overcome the structural and molecular barriers imposed by the chronic glial scar. Within this broader landscape, our findings support a complementary perspective: rather than viewing rehabilitation and regeneration as parallel or competing strategies, an early conditioning phase that preserves peripheral targets may enhance the effectiveness of later microenvironment-modifying interventions. By mitigating excitotoxicity, inflammation, and astroglial hypertrophy, BoNT/A may contribute to a more permissive lesion environment that could, in principle, synergize with pro-regenerative approaches. Crucially, BoNT/A was ineffective when administered alone during the chronic phase, failing to restore motor function or reduce tissue degeneration in the absence of EMS preconditioning, unlike when injected during the acute phase [13], [14]. This suggests that BoNT/A’s effectiveness in the chronic phase relies on the preservation of structural connectivity and cellular responsiveness, which are supported by EMS. Once these conditions are met, BoNT/A appears to act as a potent disease-modifying agent, reducing gliosis, excitotoxicity, apoptosis, and promoting oligodendrocyte survival and remyelination.

Moreover, we demonstrated that BoNT/A treatment also when administered in chronic phase, in mild/moderate contused mice, can mitigate pain-related mechanisms, significantly attenuating NeP-like behaviors. While the peripheral analgesic properties of BoNT/A are well-established [19], [44], [45], [46] our findings extend its relevance to central injury models, indicating a broader therapeutic spectrum that includes central antinociceptive activity, likely mediated by glial modulation and rebalancing of excitatory neurotransmission To elucidate the mechanisms by which BoNT/A promotes motor recovery and alleviates NeP, we demonstrate that BoNT/A induces a profound remodelling of astrocytes in the injured spinal cord, an effect that goes well beyond simple attenuation of astrogliosis. Astrocytes play a pivotal role in regulating glutamate clearance, modulating both excitatory and inhibitory neurotransmission, and maintaining overall synaptic homeostasis [47]. Our findings indicate that BoNT/A modulates injury-associated astrocytic reactivity in the chronically injured spinal cord, neurotoxic profile toward a morphology and molecular signature more compatible with homeostatic and neuroprotective states [48], [49], [50]. Rather than producing a uniform suppression of gliosis, BoNT/A acts preferentially within the lesion EPI, the region of highest metabolic burden and glutamate overload, where astrocytes typically display the most pronounced A1-like features [34], [35]. This region-specific effect suggests that BoNT/A targets the cellular compartments under maximal stress, possibly by modulating cytoskeletal dynamics and intracellular trafficking, two processes tightly linked to astrocytic hypertrophy [34].

The structural remodeling observed in BoNT/A-treated animals, characterized by compact arbors and reduced territorial expansion, is consistent with a shift away from A1-like morphology, which is defined by extensive process hypertrophy and increased GFAP polymerization. This interpretation is reinforced by molecular signatures known to distinguish astrocytic phenotypes. A1 astrocytes downregulate glutamate transporters and exacerbate inflammatory signaling [33], [34], while A2 astrocytes display enhanced EAAT1/GLAST expression, supporting synaptic function and neuronal survival [34]. The preservation of EAAT1 we observed after BoNT/A treatment aligns closely with this A2-like profile and is consistent with prior descriptions of EAAT1 as a hallmark of reparative astrocytes [34]. The modulation of glutamatergic markers further supports this interpretation. Although vGLUT1 [32] remains chronically reduced after injury [51], an established long-term consequence of excitatory circuit remodeling, BoNT/A decreased GFAP-vGLUT1 colocalization, suggesting a more selective and compartmentalized astrocyte-synapse interaction. Importantly, this reduction occurs in the context of preserved EAAT1, indicating that BoNT/A does not impair glutamate buffering but may limit maladaptive perisynaptic enwrapping typical of A1-like astrocytes. A less intrusive, more spatially confined astrocytic presence may help restore synaptic homeostasis and reduce chronic excitotoxic stress.

Together, these observations support the view that BoNT/A promotes a region-selective modulation of astrocytic structure and associated molecular markers. While the present study does not directly define astrocyte “states”, the convergent morphology- and marker-based data are consistent with a shift toward less hypertrophic, functionally supportive astrocytic profiles. This change may arise from both direct modulation of astrocytic signaling pathways and indirect effects on the progenitor cell niches that contribute to astroglial turnover, as suggested by our previous demonstration that BoNT/A enhances the expansion of Nestin⁺ populations in the injured spinal cord [13]. In the chronic phase of SCI, where gliosis becomes maladaptive and contributes to synaptic dysfunction, inflammatory persistence, and metabolic impairment, the ability of BoNT/A to remodel astrocytic structure and restore key homeostatic functions may represent a mechanism underlying its therapeutic potential.

The observed modulation of microglial cell morphology and phenotype following BoNT/A treatment points to a broader anti-inflammatory mechanism beyond astrocytic remodelling. Chronic activation of microglia has been recognized as a key driver of secondary degeneration in SCI, sustaining a proinflammatory milieu through persistent release of cytokines (e.g., IL-1β, TNF-α), reactive oxygen species, and matrix metalloproteinases [52], [53]. Importantly, this study captures a cellular landscape that remains poorly characterized in the literature, the chronic phase of SCI, both because most animal models fail to fully mirror long-term human pathology [15], [54] and because severe contusion models rarely include extended temporal analyses of glial dynamics [55]. Our data reveal an unexpectedly heterogeneous and spatially segregated glial environment in chronic SCI, where distinct microglial, astrocytic, and oligodendroglial phenotypes (discussed below) coexist in region-specific patterns. This complexity underscores the importance of studying chronic time points, where tissue remodeling, ongoing degeneration, and compensatory responses converge into a highly dynamic neuroimmune microenvironment.

In the chronic phase of SCI, microglia display substantial morphological heterogeneity, which likely reflects the intrinsic complexity of long-term neuroinflammation rather than experimental variability. This diversity of phenotypes, coexisting within the same tissue and differing across regions, may provide a more faithful representation of the heterogeneous cellular responses reported in clinical SCI. Within this framework, the trends observed in our study suggest that BoNT/A may contribute to maintaining a greater proportion of ramified, less reactive microglia, particularly in the perilesioned area, whereas saline-treated animals more frequently exhibit reactive and rod-like forms typically associated with persistent inflammatory activation.

The general pattern of morphometric analyses such as Sholl profiling is consistent with these phenotypic trends and with the expected variability of chronic SCI, where regional microenvironments strongly shape microglial structure. Similarly, changes in Iba1^+^ area likely reflect differences in phenotype composition rather than absolute changes in activation, ramified cells occupy larger territories, while reactive forms are more compact despite being more inflammatory. Overall, although subtle, these converging observations indicate that BoNT/A may modulate microglial behavior within a highly heterogeneous chronic landscape, supporting a shift toward states less associated with sustained inflammation. Crucially, the variability observed across all groups highlights a fundamental characteristic of chronic SCI and underscores the need to interpret glial responses within this inherently diverse biological context.

These effects echo BoNT/A’s known actions in peripheral inflammatory models, where it suppresses the release of proinflammatory mediators from both microglia and macrophages [12], [56]. By reducing microglial reactivity at structural and functional levels, BoNT/A may dampen the chronic inflammatory loop that impedes regeneration in SCI. These morphological changes were paralleled by a selective increase in β-actin expression in the EMS+BoNT/A group, as revealed by Western blotting analysis. Although traditionally used as a housekeeping control, actin levels are known to fluctuate in response to cytoskeletal remodelling and glial activation states [47]. The observed increase may reflect an ongoing structural reorganization of glial cytoskeleton, supporting the notion that BoNT/A exerts its effects by reprogramming glial morphology and function at both cellular and molecular levels.

Taken together with the astrocytic remodelling described above, the data suggest that BoNT/A orchestrates a coordinated reprogramming of the glial landscape, acting on both astrocytes and microglia, to mitigate chronic neuroinflammation. This dual action likely contributes to the creation of a more permissive environment for neuronal survival and synaptic preservation, reinforcing the therapeutic relevance of BoNT/A as a glia-targeting agent in SCI and other neuroinflammatory conditions. Together, these findings suggest that BoNT/A can reshape the chronic SCI environment to promote recovery by acting primarily on glial components. Unlike classical neurotrophic or anti-inflammatory drugs, BoNT/A does not target neuronal receptors or broadly suppress inflammation. Instead, it enables selective glial reprogramming and preservation of oligodendrocyte-mediated myelination, facilitating long-term improvements in tissue integrity and function.

A key outcome of BoNT/A treatment in our chronic SCI model was the preservation of neural tissue integrity, characterized by a significant reduction in apoptotic cell death and an increase in neuronal and oligodendroglia survival. TUNEL and Caspase-3 staining revealed fewer apoptotic cells in BoNT/A-treated animals, with clear evidence of protection in NeuN⁺ neurons and Olig1⁺ oligodendroglia cells. Interestingly, these effects occurred in the absence of synaptic receptor normalization: both NMDA and gamma-aminobutyric acid type A receptor (GABA-A) receptor subunit expression remained equally dysregulated across all injured groups, including BoNT/A-treated animals. This further supports the idea that BoNT/A operates upstream of direct synaptic repair mechanisms, likely through the modulation of glial-derived signals and perisynaptic homeostasis.

Interestingly, the neuroprotective effects of BoNT/A occurred despite persistent downregulation of both NMDA and GABA-A receptor subunits at 60 dpi. For GABA-A, this aligns with evidence that inhibitory failure after SCI is driven less by receptor abundance and more by interneuron loss, reduced glutamic acid decarboxylase 65/67, increased uptake, and chloride dysregulation [potassium-chloride cotransporter 2 (KCC2) downregulation/Na-K-Cl cotransporter 1 (NKCC1) upregulation]. BoNT/A likely preserves neurons and oligodendrocytes by reducing excitotoxic and glial-derived stress rather than directly reinstating GABAergic tone, suggesting potential synergy with strategies that restore inhibition (e.g., baclofen, GABA transporter inhibitors, KCC2 enhancers) [55], [56], [57], [58], [59], [60]. For NMDA receptors, chronic dysregulation reflects complex, subunit- and compartment-specific remodelling not captured by segmental Western blotting. BoNT/A dampens excitotoxicity upstream but does not normalize NMDA subunit expression, indicating that combinations with NMDA-targeted interventions [e.g., N-methyl D-aspartate receptor subtype 2B (NR2B) receptor antagonists; low-dose NMDA blockers; disruptors of the link between the postsynaptic density 95 (PSD-95) and the neuronal nitric oxide synthase (nNOS)] may further enhance functional recovery [61], [62], [63], [64].

This neuroprotection may be partly explained by BoNT/A’s capacity to reduce extracellular glutamate [65], [66], [67] and attenuate excitotoxic signalling, which are key contributors to secondary degeneration in SCI. By dampening glial reactivity and restoring perisynaptic organization, BoNT/A likely promotes a permissive environment for synaptic reorganization, axonal sprouting, and endogenous repair processes. These effects may help initiate functional improvements such as motor recovery, which has been shown to correlate with reduced neuronal apoptosis and glutamate spillover in chronic SCI [68], [69].

One of the most striking outcomes was the enhanced preservation of oligodendroglial lineage cells and increased myelin protein expression in BoNT/A-treated animals. In *ex-vivo*, we observed a higher density of Olig1⁺ cells and significant upregulation of MBP. An additional, treatment-independent observation emerging from our OLIG1 analyses concerns the marked phenotypic heterogeneity of oligodendrocyte lineage cells across different anatomical regions of the chronically injured spinal cord. OLIG1-expressing cells adopt distinct morphologies in the EPI, perilesional areas, and scar tissue, a finding that aligns with developmental and injury-related dynamics previously described in the literature [70]. During normal CNS development, OLIG1 undergoes a well-characterized shift from a nuclear to a cytoplasmic localization, a transition required for oligodendrocyte maturation and myelin membrane expansion [38]. OPCs also display high morphological plasticity, transitioning from elongated bipolar shapes during early migration to increasingly branched, ramified morphologies as they mature and interact with axons [70], [71]. In adulthood, OPCs retain a surveying phenotype with stable but dynamic processes, yet upon CNS injury, they may either reacquire a hypertrophic, NG2-upregulated profile or revert to a bipolar motile phenotype to facilitate recruitment toward the lesion core [70]. *In vitro*, BoNT/A promoted the differentiation of OPCs into mature oligodendrocytes, increased process complexity, and elevated expression of myelin-related markers such as MBP, GalC, PLP, and MOG. These effects were observed under both proliferative and differentiating conditions, indicating that BoNT/A acts even in the absence of extrinsic pro-differentiation cues. One plausible explanation for this sensitivity lies in the differential expression and role of SNARE proteins along the oligodendrocyte lineage. While SNAP25 is typically regarded as a neuron-specific SNARE, its expression has now been confirmed in human oligodendrocytes [36]. Our findings of cl-SNAP25 signal in PLP⁺ and MOG⁺ cells, particularly in BoNT/A-treated non-differentiated cultures, suggest that SNAP25 is not only present but also accessible to BoNT/A enzymatic activity in the oligodendroglia lineage.

Notably, this effect was most prominent in NG2⁺ OPCs under non-differentiating conditions. The highest levels of cl-SNAP25 in non-differentiated cells suggest that BoNT/A preferentially acts on immature cells, possibly because of higher endocytic activity, differential SNARE expression, or increased expression of BoNT/A receptor (synaptic vesicle protein 2). These hypotheses are supported by studies indicating that OPCs, particularly NG2⁺ cells, display a high degree of synaptic interaction with glutamatergic neurons and possess functional postsynaptic specializations, including vesicle release and uptake machinery [72], [73], [74]. Notably, NG2⁺ cells are not merely precursors but are now recognized as active regulators of CNS plasticity. Their ability to receive synaptic input, respond to neurotransmission, and regulate axon-glia interaction positions them as dynamic participants in injury and repair [75], [76]. The fact that BoNT/A has a more pronounced effect on these cells in a non-differentiated state raises the possibility that early-stage OPCs are “primed” for BoNT/A-mediated modulation, perhaps via SNARE-dependent control of metabolic vesicle delivery or local calcium buffering, as suggested for early myelin assembly [76].

These findings provide a compelling rationale to further explore how BoNT/A interfaces with the unique synaptic-like physiology of NG2⁺ OPCs, which, unlike mature oligodendrocytes, are capable of bidirectional communication with neurons and other glia [77], [78], [79]. In this framework, BoNT/A may operate not only as a blocker of excitatory stress but also as a modulator of glial vesicle signalling, tipping the balance toward differentiation and myelination. In addition, we observed a stronger response to BoNT/A in terms of branching complexity in the non-differentiation condition, measured in MBP and MOG stained, and process length, in the differentiated condition. Of note, our findings seem in contrast with those of Chacon-De-La-Rocha *et al.* [80], who reported a decrease in NG2⁺ OPC complexity after intrahippocampal BoNT/A administration. However, their use of a much higher BoNT/A concentration (1 nmol/L vs. 10 pmol/L) and their targeting of a non-lesioned, synaptically dense brain region likely contribute to this discrepancy. Nonetheless, their study supports the notion that BoNT/A can act directly on OPCs *in vivo*, further validating our mechanistic hypothesis.

Taken together, these results suggest that BoNT/A action on oligodendrocyte lineage cells may operate through a dual mechanism. On one side, our *in vitro* experiments with purified OPCs provide evidence for a direct effect, supported by the presence of cl-SNAP25 in PLP⁺/MOG⁺ cells and by the enhanced differentiation observed under both proliferative and differentiating conditions. On the other side, in the *ex vivo* and *in vivo* context, BoNT/A may also act indirectly by shaping the spinal cord microenvironment. The reduction of excitotoxicity and proinflammatory mediators, as shown in our study, likely decreases secondary cell death and creates a permissive milieu that supports survival and maturation of oligodendrocyte lineage cells. Consistently, BoNT/A-treated animals displayed increased oligodendroglial preservation in parallel with reduced apoptosis. We therefore propose that BoNT/A enhances remyelination through a synergistic interplay between direct OPC modulation and indirect neuroprotective effects within the injured tissue

This study also underscores the importance of context and timing in BoNT/A efficacy (all results are summarized in a schematic representation in **Additional file 1:** Fig. S12). Only when combined with early rehabilitation (EMS) did BoNT/A achieve meaningful functional outcomes. This has important implications for clinical trial design, indicating that BoNT/A may be most effective in patients undergoing or having completed structured neuromuscular reconditioning. The observed effects on glia and myelin further support the rationale for targeting chronic neuroinflammation and demyelination in SCI and related neurodegenerative disorders.

The current research, along with other scientific evidence published by our team [13], [14], [15], strongly supports future steps toward translating these findings into an upcoming clinical phase of BoNT/A use in SCI patients. This study is part of a broader translational R&D program aimed at repurposing BoNT/A, following a strategic development plan aligned with European regulatory guidelines. Our approach is also consistent with the objectives of the pilot project “Repurposing of authorised medicines: pilot to support not-for-profit organisations and academia”, launched by the European Medicines Agency and the Heads of Medicines Agencies [81]. The outcomes of this initiative, expected to be published in 2025, will contribute to the development and implementation of a formal repurposing framework, which will guide our future studies on BoNT/A as a repurposed therapeutic agent. To enable clinical translation, we are pursuing a targeted development pathway that includes early dialogue with regulatory authorities and the preparation of a comprehensive preclinical dossier to support the initiation of a clinical trial of BoNT/A in SCI patients. On this basis and by bridging functional rehabilitation and targeted neurobiological modulation, this innovative approach may offer a novel therapeutic avenue where few options currently exist.

### 4.1 Limitations, future directions, and clinical perspectives

This study demonstrates that BoNT/A can exert robust neuroprotective and pro-regenerative effects in chronic SCI, preserving neurons and oligodendrocytes, reducing apoptosis, and attenuating astrogliosis and microglial activation. At the same time, several aspects deserve further clarification to strengthen both the mechanistic understanding and the translational potential of this approach.

One important aspect concerns the use of female mice. This choice was deliberate, as previously validated in our model [15]: male mice often show a degree of spontaneous motor recovery even in the absence of treatment, a feature with little translational relevance given that patients with severe SCI rarely recover motor function spontaneously. Female mice, by contrast, provide a more stable and clinically relevant representation of motor outcome. While this enhances translational fidelity, it also raises the need to investigate sex-specific responses more systematically in future studies, especially as sex differences in neuroinflammation, metabolism, and regeneration are increasingly recognized.

A further considerationrelates to the dosing regimen and follow-up window. We tested a single dose of BoNT/A, chosen in line with its long-lasting biological activity. In humans, therapeutic efficacy typically extends for 2–4 months and can last up to 6 months, consistent with toxin turnover and immune clearance. As preliminary indications of dosing, the dose of 15 pg/mouse of BoNT/A used in this study can be considered as animal no-observed-adverse-effect level (NOAEL). Under this assumption, considering an average weight of mice of 40 g, our dose corresponds to a mouse NOAEL dose of 0.375×10^–6^ mg/kg and, considering 60 kg as human standard weight, to a human equivalent dose (HED) of 0.033×10^–6^ mg/kg. Based on the amount of toxin contained in the commercial toxins [82] this HED correspond to 4.5 U Botox/kg, 5 U Dysport/kg and 7.5 U Xeomin/kg, or in other way to 270 U Botox, 300 U Dysport and 450 U Xeomin in a human weighing 60 kg. By reducing these values by a factor of 10 for safety reasons, doses of 27 U Botox, 30 U Dysport, and 45 U Xeomin are obtained, which are doses compatible with the doses currently used in clinic for single administration.

Our 60-day follow-up in mice captures the mid-range of this therapeutic window, but longer time points will be needed to assess whether the beneficial effects persist or decline. Optimizing dosing, timing, and the possibility of repeated administrations will be essential steps toward clinical translation.

Mechanistically, while BoNT/A clearly preserved astrocytic and oligodendroglia populations, the nature of these effects remains to be clarified. The increased astrocytic density associated with reduced hypertrophy suggests a “scar-modulating” rather than “scar-forming” phenotype. Whether this results from proliferation, migration, or phenotypic reprogramming requires targeted analyses, including proliferation markers and lineage tracing as well as changes in the expression of proinflammatory mediators, such as cytokines and chemokines, to determine whether the morphological normalization of astrocytes and microglia observed after BoNT/A treatment corresponds to a functional shift toward a less inflammatory state. Similarly, the preservation of oligodendrocyte lineage cells may arise from both direct BoNT/A actions, supported by our *in vitro* data, and indirect modulation of the microenvironment through reduced excitotoxicity and inflammation. Disentangling these contributions will be a priority for future mechanistic work.

An additional limitation is that functional receptor balance was not restored: NMDA and GABA-A receptor subunits remained dysregulated across groups. This finding highlights the complexity of chronic excitatory/inhibitory remodelling after SCI and suggests that BoNT/A acts upstream, reducing excitotoxicity and inflammatory cascades without directly resetting receptor expression or chloride homeostasis. Future studies should therefore include subunit- and region-specific analyses, assessment of KCC2/NKCC1 expression, and functional electrophysiology to evaluate inhibitory and excitatory currents. Importantly, this opens the door to combination strategies, in which BoNT/A could be paired with interventions that directly reinforce GABAergic inhibition or normalize NMDA signalling, maximizing neuroprotection and functional recovery.

Overall, although BoNT/A preserved structural features such as myelination and neuromuscular junctions, functional readouts were not included. Electrophysiological measures of conduction velocity, synaptic efficacy, and network activity will be required to confirm that the structural benefits translate into improved functional connectivity.

Altogether, these results support BoNT/A as a candidate therapeutic in chronic SCI, but they also delineate a roadmap for the next steps: inclusion of both sexes, longer follow-up, optimization of dosing schedules, mechanistic dissection of glial and oligodendroglial responses, and combinatorial approaches that integrate BoNT/A with other pro-regenerative interventions. From a clinical perspective, these directions are particularly relevant, as they mirror ongoing challenges in SCI rehabilitation, sex-specific variability, chronicity of neuroinflammation, limited durability of interventions, and the need for multimodal strategies. By addressing these issues, the translational path for BoNT/A in SCI could move closer to clinical trial readiness

## 5 Conclusions

This study demonstrates that BoNT/A, when applied in a context of preserved neuromuscular integrity via electrical stimulation, can exert significant neuroprotective and pro-regenerative effects in a murine model of chronic SCI. BoNT/A modulates the spinal environment by reshaping astrocytic and microglial reactivity, reducing excitotoxic signalling, preserving oligodendrocyte lineage cells, and promoting remyelination. These actions appear to involve both direct effects on glial cells and indirect modulation of the extracellular milieu. Our results highlight BoNT/A as a unique therapeutic candidate capable of engaging glial pathways traditionally overlooked in SCI pharmacology. The findings set the stage for mechanistic studies and clinical translation, and advocate for a paradigm shift in the treatment of chronic SCI, from symptomatic management toward glia-focused repair strategies.

## Abbreviations

BMS: Basso Mouse Scale;

BoNT/A: Botulinum neurotoxin type A;

BTX: Bungarotoxin;

Cd11b: Cluster of differentiation 11b;

CNS: Central nervous system;

Col1a1: Collagen type I alpha 1;

CSA: Cross-sectional area;

DAPI: 4′,6-diamidino-2-phenylindole;

DH: Dorsal horn;

DIFF: Cell differentiation;

EAAT1/GLAST: Excitatory amino acid transporter 1;

EMS: Electrical muscle stimulation;

EPI: Epicenter;

FBXO32: F-box protein 32 (also known as MAFbx/Atrogin-1);

GA: Gastrocnemius;

GABA-A: Gamma-aminobutyric acid type A receptor;

GalC: Galactocerebroside C;

GAPDH: Glyceraldehyde-3-phosphate dehydrogenase;

GFAP: Glial fibrillary acidic protein;

GPC: Glial precursor cell;

HED: Human equivalent dose;

Iba1: Ionized calcium binding adaptor moleclule 1;

IQR: Interquartile range;

KCC2: Potassium-chloride cotransporter 2;

MBP: Myelin basic protein;

micro-CT: Micro-computed tomography;

MOG: Myelin oligodendrocyte glycoprotein;

NeP: Neuropathic pain;

NeuN: Neuron-specific nuclear protein;

NG2: Neuron-glial antigen 2;

NMDA: N-methyl-D-aspartate;

NKCC1: Na-K-Cl cotransporter 1;

NOAEL: No-observed-adverse-effect level;

NO DIFF: Non-differentiating (proliferative) condition;

Olig1: Oligodendrocyte lineage transcription factor 1;

OPC: Oligodendrocyte precursor cell;

PLP: Proteolipid protein (PLP1);

SCAR: Scar region;

SCI: Spinal cord injury;

SEM: Standard error of the mean;

SRH: Scheirer-Ray-Hare test;

SNAP25: Synaptosomal-associated protein 25;

Syn: Synaptophysin

cl-SNAP25: Cleaved synaptosomal-associated protein 25;

SNARE: Soluble NSF attachment protein receptor;

TRIM63: Tripartite motif containing 63 (MuRF1);

TUNEL: Terminal deoxynucleotidyl transferase dUTP nick end labeling;

vGLUT1: Vesicular glutamate transporter 1;

VH: Ventral horn;

WHO: World Health Organization.

## Ethics approval and consent to participate

All procedures were in strict accordance with the European and Italian National law (DLGs n.26 of 04/03/2014, application of the European Communities Council Directive 2010/63/UE) on the use of animals for research (Italian Ministry of Health - protocol code 122/2019PR) and with the guidelines of the Committee for Research and Ethical Issues of IASP.

## Authors’ contributions

SM and VM contributed to the conceptualization of the study. Methodology was developed by SM, LM, MC, VM, FDS, FDA, CP, FP, SL, OR, and GS Investigation was carried out by SM, SL, GR, VR, LAP, CP, VM, VV, FDS, LM, SA, ADE, and RM. Statistical analyses were performed by SM, VM, LM, and MC. SM and VM wrote the original draft. Writing review and editing was performed by SM, SL, OR, LM, and GS. Funding acquisition was led by SM and LM. All authors read and approved the final manuscript.

## Funding

This work was supported by the 2021 SCI-BTXA PoC MISE - Proof of Concept – Italian Ministry of Economic Development (to Sara Marinelli), the French Muscular Dystrophy Association (AFM-Telethon) Research Grant (#24349), and the “Progetti di Ricerca di Rilevante Interesse Nazionale” (PRIN) 2022 (2022WN338R, to Luca Madaro).

## Data Availability

The datasets generated and/or analysed during the current study are available from the corresponding author on reasonable request.

## Footnotes

Appendix A Supplementary data associated with this article can be found in the online version at doi:10.1016/j.mmr.2026.100049.
