# Supplementary material for: A translational preclinical strategy for chronic spinal cord injury: neuroprotective and regenerative potential of botulinum neurotoxin type A combined with muscle atrophy prevention via electrostimulation
Source: Mil Med Res. 2026 Jun 27;13(1):100049. doi: 10.1016/j.mmr.2026.100049 (PMC13321029; doi:10.1016/j.mmr.2026.100049)
Supplement: Supplementary file 3 — Supplementary material [file mmc1.pdf]

Methods

Randomization, allocation concealment, and blinding

All mice assigned to electrical muscle stimulation (EMS) underwent rehabilitation and behavioral testing by Experimenter #1. At the end of the EMS phase, Experimenter #2 prepared either botulinum neurotoxin type A (BoNT/A) or vehicle solutions and labelled each vial with an alphanumeric code (distinct code per vial); the content was concealed from all other operators. Subsequently, Experimenter #3, who had not participated in the rehabilitation/testing phase, randomly selected and injected the solution from vial "1" or "2" under blinded conditions. Following injections, animals, now randomly allocated to treatment, were re-tested and harvested by Experimenter #1, still blinded to group identity. Harvested tissues were then distributed to collaborators for the various assays (histology, imaging, Western blotting, quantitative PCR), each sample identified only by its code; codes were revealed after data lock and statistical analysis. This three-operator workflow ensured allocation concealment, blinding of outcome assessors, and randomization of treatment assignment.

Surgery

Postoperative care included housing under red light for the first 24 h, subcutaneous injection of betamethasone (1 mg/kg), and provision of water-based gel and softened food in the cage to support hydration and nutritional intake. Bladders were manually expressed twice daily until spontaneous voiding was restored.

Potential outliers were identified through analysis of impact parameters recorded by the PinPoint software (Leica Geossystem S.p.A., Cornegliano Laudense, LO, Italy). Behavioral and observational criteria were also used to assess variability in injury severity across groups. It is important to note that not all animals subjected to "severe" injury parameters developed complete hindlimb paralysis, likely due to individual differences in spinal cord compression caused by the underlying vertebral structure.

Electrical muscle stimulation

Regarding the EMS treatment, a small number of severely injured animals (n=6) were assigned to the non-stimulated group as the control group. This decision was made in accordance with the 3Rs principle (Replacement, Reduction, Refinement), as the spinal cord injury (SCI) protocol induces a high degree of suffering and is associated with prolonged impairment over the 60-day experimental period. Based on our previously published data [1,2], animals subjected to severe SCI without EMS show no spontaneous functional recovery and, in fact, exhibit progressive deterioration in general health and behavior. Therefore, only a minimal number of animals were allocated to untreated conditions to reduce unnecessary distress while preserving scientific validity.

In preliminary experiments, we also tested a passive locomotor rehabilitation protocol using treadmill training (**Additional file 1: Fig. S2** for details). This approach was initially chosen with the rationale of exploiting residual spinal locomotor circuits (central pattern generators) [3] that might be recruited by repetitive stepping-like movements. However, despite the progressive increase in training speed and duration, treadmill training failed to promote motor recovery in severely injured animals, as Basso Mouse Scale (BMS) scores remained unchanged over the 60-day observation period. Based on this lack of efficacy, we focused on EMS as a more effective strategy to prevent muscle atrophy and to create a permissive environment for subsequent BoNT/A treatment.

Three-dimensional reconstruction and skeletal segmentation

Projection data were reconstructed using the proprietary MILabs software (MILabs, Houten, The Netherlands), producing volumetric datasets with isotropic voxel size of 80 µm. The reconstructed images were exported in DICOM format and analysed using Imalytics Preclinical image analysis software (Gremse-IT GmbH, Germany). Grayscale values were calibrated to Hounsfield units to allow standardized interpretation. Three-dimensional segmentation and rendering of the spinal cord were subsequently performed to isolate and quantify vertebral and adjacent skeletal structures within the thoracolumbar region.

Confocal microscopy and quantification of immunoresponsivity

To compare cell counts obtained from 63× and 40× objectives on an equivalent field basis, 63× counts were scaled to match the larger tissue area captured at 40×. Because the 40× field of view is 2.476-fold larger than the 63× field (150,156 µm² vs. 60,657 µm²), 63× counts were corrected using:

$$N_{\text{scaled}(40\times)} = N_{63\times} \times 2.476$$

This area-based upscaling allows direct comparison between datasets acquired at different magnifications.

Neuron-specific nuclear protein (NeuN)$^+$ and terminal deoxynucleotidyl transferase dUTP nick end labeling (TUNEL)$^+$ nuclei were automatically counted. TUNEL$^+$ nuclei were quantified using ImageJ. After background subtraction and signal saturation to enhance nuclear circularity, images were converted to binary masks. Cell detection was then performed with the “Analyze Particles” tool, applying a minimum size threshold of 50 pixels to prevent residual background artifacts or very small nonspecific elements from being counted as cells (*n* per each experiment/group and number of slices utilized are reported in the figure legend), and group means were subsequently calculated.

Astrocytic and microglia distribution and morphology were analysed on confocal images of glial fibrillary acidic protein (GFAP) or ionized calcium binding adaptor moleclule 1 (Iba1)/cluster of differentiation 11b (Cd11b), respectively, immunofluorescence acquired from spinal cord sections encompassing the lesion epicenter (T9–T11), perilesional regions (T7–T8 and T12–T13), and the

epicenter-scar area. Each image was converted to 8-bit grayscale and binarized using the Otsu thresholding algorithm to generate a binary mask.

The total $GFAP^+$ or $Iba1^+/Cd11b^+$ area was measured using the "Analyze Particles" function, expressing the value in pixels. The size and circularity parameters were set to discriminate cell bodies from processes divided from the soma. Specifically, a range of 200–∞ pixels (circularity 0–1) was used to quantify the total area occupied by $GFAP^+$ elements, including detached processes, while a more restrictive range of 400–∞ pixels was applied to estimate the number of astrocytic cell bodies. For microglia, since its small size, a range of 50–∞ pixels (circularity 0–1) was used to quantify the total area occupied by $Iba1^+/Cd11b^+$ elements, including detached processes, while a more restrictive range of 100–∞ pixels was applied to estimate the number of microglia cell bodies.

In the scar region, characterized by a dense and irregular GFAP network or cystic cavities devoid of cell bodies, only the total $GFAP^+$ area was measured. Representative images showing the original acquisition, binary mask, and segmented overlays are reported in the **Additional file 1: Fig. S3**.

Fluorescence intensity for caspase-3 and oligodendrocyte lineage transcription factor 1 (Olig1) was quantified using the RGB method, which converts red, green, and blue pixel values to brightness levels. In addition, signal quantification and colocalization within defined regions of interest were assessed using integrated density measurements and Manders' colocalization coefficient.

RNA analysis by quantitative PCR

Total RNA was extracted from frozen gastrocnemius (GA) muscle tissue using Tri-reagent (Zymo Research) following the manufacturer's instructions. RNA concentration and purity were assessed with a NanoDrop ONEc spectrophotometer (Thermo Scientific). First-strand cDNA was synthesized from the extracted RNA using the PrimeScript RT Reagent Kit with gDNA Eraser (TaKaRa) according to the manufacturer's protocol. Quantitative real-time PCR was performed on a QuantStudio 7 Flex system (Applied Biosystems, Thermo Fisher Scientific, Waltham, MA, USA) using the ExcelTaq 2X Fast Q-PCR Master Mix (SYBR, ROX) (Smobio, 7 BioScience, Neunburg am Rhein, Germany). The detailed list of primers used is described in **Additional file 1: Table S1**.

Muscle immunofluorescence staining

GA muscle cryosections (10 μm) were fixed in 4% paraformaldehyde (PFA; MilliporeSigma, Darmstadt, Germany; P6148) for 10 min or permeabilized with 100% acetone for 1 min at room temperature. Sections were blocked for 1 h in phosphate-buffered saline (PBS) containing 4% bovine serum albumin (BSA; MilliporeSigma, Darmstadt, Germany; A7030-100G). Primary antibodies were diluted in block solution and applied overnight at 4 °C: anti-synaptophysin (Syn), anti-laminin, and anti-caveolin-3 as specified in **Additional file 1: Table S2**. After washing with PBS, sections were incubated with species-appropriate secondary (**Additional file 1: Table S2**). Acetylcholine receptors

(AChRs) were visualized using fluorescently labelled bungarotoxin (BTX) (**Additional file 1: Table S2**). Nuclear staining was performed with 4',6-diamidino-2-phenylindole (DAPI) in PBS for 5 min. Finally, sections were washed in PBS and mounted in glycerol (3:1 in PBS). Each muscle was separately analysed. Images were acquired using a Zeiss confocal microscope and processed with ImageJ software.

Sirius red staining

GA muscle cryosections were fixed for 1 h at 56 °C in Bouin's solution (Sigma-Aldrich, Darmstadt, Germany; Cat #HT10132), then stained for 1 h in Picro-Sirius red solution (Direct Red 80 Cat #365548; Sigma-Aldrich, Darmstadt, Germany) protected from light. Sections were briefly washed in acidified water (0.5% vol/vol), dehydrated in 100% ethanol, cleared in 100% toluene, and mounted with EUKITT mounting medium (Sigma-Aldrich, Darmstadt, Germany; Cat #03989). Images were acquired with a Zeiss Imager.A2 microscope.

Haematoxylin and eosin (H&E) staining

GA sections were fixed in 4% PFA for 10 min, washed in PBS, then stained with haematoxylin (Sigma-Aldrich, Darmstadt, Germany; Cat #HHS32) for 12 min and eosin (Sigma-Aldrich, Darmstadt, Germany; Cat #HT110332) for 30 s. Sections were dehydrated in ethanol and mounted with EUKITT mounting medium (Sigma-Aldrich, Darmstadt, Germany; Cat #03989).

Immunohistochemistry of spinal cord tissues

Sixty days post-SCI, three or four mice from each experimental group (except for the non-stimulated, untreated SCI group, for which only two/three animals were available) were sacrificed for immunohistochemistry and perfused with saline 0.9% followed by 4% PFA in PBS (pH 7.4). Spinal cords were then collected and kept for 48 h in PFA at 4 °C, then cryo-protected overnight in sucrose dissolved at 30% in PBS 1× and finally cryopreserved at –80 °C. Slicing of the spinal cord was carried out by embedding the tissues in Tissue-Tek OCT (Sakura, Torrance, CA, USA) and sections of 40 μm thickness were collected. For double immunofluorescence staining, different sections were incubated for 48 h at room temperature with primary antibodies (**Additional file 1: Table S2**) in Triton 0.3%. Sections were then washed in PBS and incubated for 2 h, at room temperature, with secondary antibodies (**Additional file 1: Table S2**). Sections were again washed in PBS and incubated for 10 min with Bisbenzimide (Hoechst 33258, 1:1000, Jackson ImmunoResearch, Ely, UK) to stain nuclei. Sections were finally mounted on glass slides with glycerol 3:1 in PBS.

Western blotting analysis

Total proteins were extracted from spinal cord in RIPA buffer [50 mmol/L Tris-HCl pH 8.0; 150 mmol/L NaCl; 1 mmol/L EDTA pH 8.0; 1% Triton; 0.1% sodium dodecyl sulfate (SDS); 1% sodium

deoxycholate (NaDOC) supplemented with protease and phosphatase inhibitors (5 mg/ml Aprotinin; 5 mg/ml Leupeptin; 5 mg/ml Pepstatin; 1 mmol/L phenylmethansolfonyl fluorure (PMSF); 10 mmol/L NaF; 200 mmol/L Na_3VO_4; 500 mmol/L β-glycerophosphate) by manual mincing of tissues with a plastic pestle, followed by sonication (microtip, power 2, 10+10 s). Then the samples were rocked on a wheel (20–30 min, +4 °C) and centrifuged (12,000 rpm, 15 min, +4 °C), to remove the tissue debris and stored at –80 °C. The protein concentration was detected by measuring 595 nm absorbance after staining with Bradford dye (BioRad, Segrate, MI, Italy; 5000006). 10–25 μg of proteins was run on polyacrylamide gels and transferred electrophoretically to 0.45 μm nitrocellulose membranes (BioRad, Segrate MI, Italy; 162-0115) using the wet system from Bio-Rad. After blocking with 5% non-fat dry milk for 1 h, membranes were incubated at +4 °C overnight with the primary antibodies: NMDA receptor subunit ε2 (NMDAε2) receptor (1:1000, ELK Biotechnology, Sugar Land TX, USA; ES5659), gamma-aminobutyric acid type A receptor subunit α2 (GABA-Rα2) (1:1000, ELK Biotechnology, Sugar Land TX, USA; EA285), myelin basic protein (MBP) (1 μg/ml; Abcam, Amsterdam, The Netherlands; ab62631), GAPDH (1:5000, Invitrogen, Monza MB, Italy; MA5-15738) and β-actin (1:5000, Invitrogen, Monza MB, Italy; MA1-744); anti-GFAP (1 μg/ml, Invitrogen, Monza MB, Italy; #14989282 Mouse Monoclonal); anti-vesicular glutamate transporter 1 (vGLUT1) (1:1000, Synaptic Systems, Goettingen, Germany; #135 302 Rabbit Polyclonal); anti-excitatory amino acid transporter 1 (EAAT1) (1:5000, MilliporeSigma, Darmstadt, Gemrany; #AB1782 Guinea Pig Polyclonal).

After three washes with PBS 0.01% Tween, membranes were incubated with secondary antibodies (donkey anti-mouse IgG-HRP 1:10,000, Jackson ImmunoResearch Europe, Ely, UK; 715-035-150; donkey anti-rabbit IgG-HRP 1:10,000, Jackson ImmunoResearch Europe, Ely, UK711-035-152; anti Guinea Pig: MilliporeSigma, Darmstadt, Germany; #AP193P 1:5000) for 1 h at room temperature and then washed with PBS 0.01% Tween. Immunoreactivity was determined using the enhanced chemiluminescence luminol reaction and revealed by Chemi-Doc Imaging System (BioRad, Segrate MI, Italy). Densitometric analysis was performed using ImageJ software.

Immunofluorescence staining of cells

OPCs and differentiating oligodendrocytes were washed with PBS and fixed using 4% PFA for 15 min, followed by permeabilization with PBS+0.1% Triton X-100, blocked in blocking buffer (3% BSA-BSA, in PBS), and subjected to incubation overnight at 4 °C with primary antibody. Then, the cells were washed three times for 5 min with PBS and incubated with fluorescence-labeled secondary antibody in the dark at 37 °C for 30–45 min. After washing three times with PBS, cells were incubated with DAPI solution (3 ng/ml) for 5 min, rinsed with PBS, and coverslips were mounted with fluoromount (Sigma Aldrich, Darmstadt, Germany) on microscope slides. The list of primary and secondary antibodies is reported in **Additional file 1: Table S2**.

Table S1 Gene-specific primer sets used for real-time PCR

Gene	Forward (5'–3')	Reverse (5'–3')
ActB	CACTGTCGAGTCGCGTCC	TCATCCATGGCGAACTGGTG
Fbxo32	CTCAGAGAGGCAGATTCGCA	GGTGACCCCATACTGCTCTC
Trim63	ACCACAGAGGGTAAAGAAGAACA	GCAGAGAGAAGACACACTTCCC
Col1a1	CGATGGATTCCCGTTCGAGT	GAGGCCTCGGTGGACATTAG

ActB. β-actin protein; Fbxo32. F-box protein 32 (also known as MAFbx/Atrogin-1); Trim63. Tripartite motif containing 63 (MuRF1); Col1a1. Collagen type I alpha 1

Table S2 List of primary and secondary antibodies

Antibody	Species	Product	Marker
Primary antibodies			
Anti-synaptophysin	Rabbit polyclonal	1:300, Invitrogen, Thermo Fisher Scientific, Monza, MB, Italy (PA1-1043)	Presynaptic vesicles
Anti-laminin	Rabbit polyclonal	1:200, Sigma Aldrich, Darmstadt, Germany (L9393)	Basal lamina
Anti-caveolin-3	Mouse monoclonal	1:1000, BD Transduction Laboratories, Milano, MI, Italy (610420)	Transmembrane protein localizes to non-clathrin membrane invaginations
Alpha-BTX	BTX conjugated Alexa Fluor 594	1:200, Invitrogen, Thermo Fisher Scientific, Monza, MB, Italy (B13423)	Acetylcholine receptors
Anti-GFAP	Mouse monoclonal anti-GFAP;<br>Rabbit anti-GFAP	1:100, Sigma-Aldrich, Darmstadt, Germany (G3893);<br>Genemed, San Francisco, CA, USA (60-0032)	Astrocyte
Anti-vGLUT1	Guinea pig polyclonal anti-vGLUT1	1:200, Millipore, Darmstadt, Germany (AB5905)	vGLUT1
Anti-Iba1	Rabbit monoclonal anti-Iba1	1:200, Abcam, Amsterdam, The Netherlands (EPR16588)	Microglia
Anti-Cd11b	Monoclonal mouse anti-rat Cd11b	1:100, BioRad, Segrate MI, Italy (MCA619R)	Microglia
Anti-NeuN	Mouse monoclonal anti-NeuN	1:100, Millipore, Darmstadt, Germany (Mab377)	Neuron
Anti-caspase-3	Rabbit polyclonal anti-caspase-3	1:100, Cell signaling, Leiden, The Netherlands (9662)	Apoptosis
Anti-Olig1	Mouse monoclonal anti-Olig1	1:100, Santa Cruz, Heidelberg, Germany (sc-166256)	Oligodendrocyte
Anti-cl-SNAP25	Rabbit polyclonal	1:200, produced in the lab of Prof. Ornella Rossetto, University of Padova, Italy	cl-SNAP25
Anti-MBP	Mouse monoclonal anti-MBP	1:100, Abcam, Amsterdam, The Netherlands (MBP101)	Myelin, mature oligodendrocyte
Anti-GalC	Mouse anti-GalC, clone mGalC	1:200, Millipore, Darmstadt, Germany (CS204476)	Pre-immature oligodendrocyte
Anti-NG2	Rabbit anti-NG2 chondroitin sulfate proteoglycan	1:200, Millipore, Darmstadt, Germany (CS204510)	Immature, migrating and proliferating OPC
Anti-MOG	Mouse anti-MOG	1:200, Millipore, Dramstadt, Germany (CS204477)	Myelin, mature oligodendrocyte
Anti-PLP1	Mouse anti-PLP1, C-terminus, clone PLPC1	1:200, Millipore, Darmstadt, Germany (CS204475)	Immature, mature oligodendrocyte

Antibody	Species	Product	Marker
TUNEL assay	Click-iT Tunel Alexa Fluor (488, 549, 647) imaging assay	Invitrogen, Thermo Fisher Scientific, Monza, MB, Italy (C10245, C10246, C10247)	Cell apoptosis
Secondary antibodies			
Alexa Fluor 488	Goat anti-mouse; Donkey anti-mouse	Invitrogen, Thermo Fisher Scientific, Monza, MB, Italy (A-11001); 1:100, Jackson ImmunoResearch, Ely, UK (715-545-150)	Green
Alexa Fluor 594	Goat anti-rabbit	Invitrogen, Thermo Fisher Scientific, Monza, MB, Italy (A-11012)	Red
Alexa Fluor 647	Goat anti-mouse	Invitrogen, Thermo Fisher Scientific, Monza, MB, Italy (A-21235)	Far Red
FITC	Rat anti-mouse; Goat anti-rabbit	1:100, Jackson ImmunoResearch, Ely, UK (212-095-168); 1:100, SantaCruz, Heidelberg, Germany (sc-2012)	Green
TRITC	Goat anti-rabbit; Goat anti-guinea pig	1:100, Jackson ImmunoResearch, Ely, UK (111-025-003); 1:100, Jackson ImmunoResearch, Ely, UK (106-025-003)	Red
Hoechst	BisBenzimide H 33342	1:1000, Sigma Aldrich, Darmstadt, Germany (14533)	Blue
DAPI	Nuclear counterstains	1:1000, Thermo Fisher Scientific, Monza, MB, Italy (D1306)	Blue

BTX. Bungarotoxin; GFAP. Glial fibrillary acidic protein; vGLUT1. Vesicular glutamate transporter 1; Iba1. Ionized calcium binding adaptor molecule 1; CD11b. Cluster of differentiation 11b; NeuN. Neuron-specific nuclear protein; SNAP25. Synaptosomal-associated protein 25; cl-SNAP25. Cleaved synaptosomal-associated protein 25; MBP. Myelin basic protein; GalC. Galactocerebroside C; NG2. Neuron-glial antigen 2; MOG. Myelin oligodendrocyte glycoprotein; PLP1. Proteolipid protein 1; OPC. Oligodendrocyte precursor cell; TUNEL. Terminal deoxynucleotidyl transferase dUTP nick end labeling; FITC. Fluorescein isothiocyanate; TRITC. Tetramethylrhodamine isothiocyanate; DAPI. 4',6-diamidino-2-phenylindole; Olig1. Oligodendrocyte lineage transcription factor 1

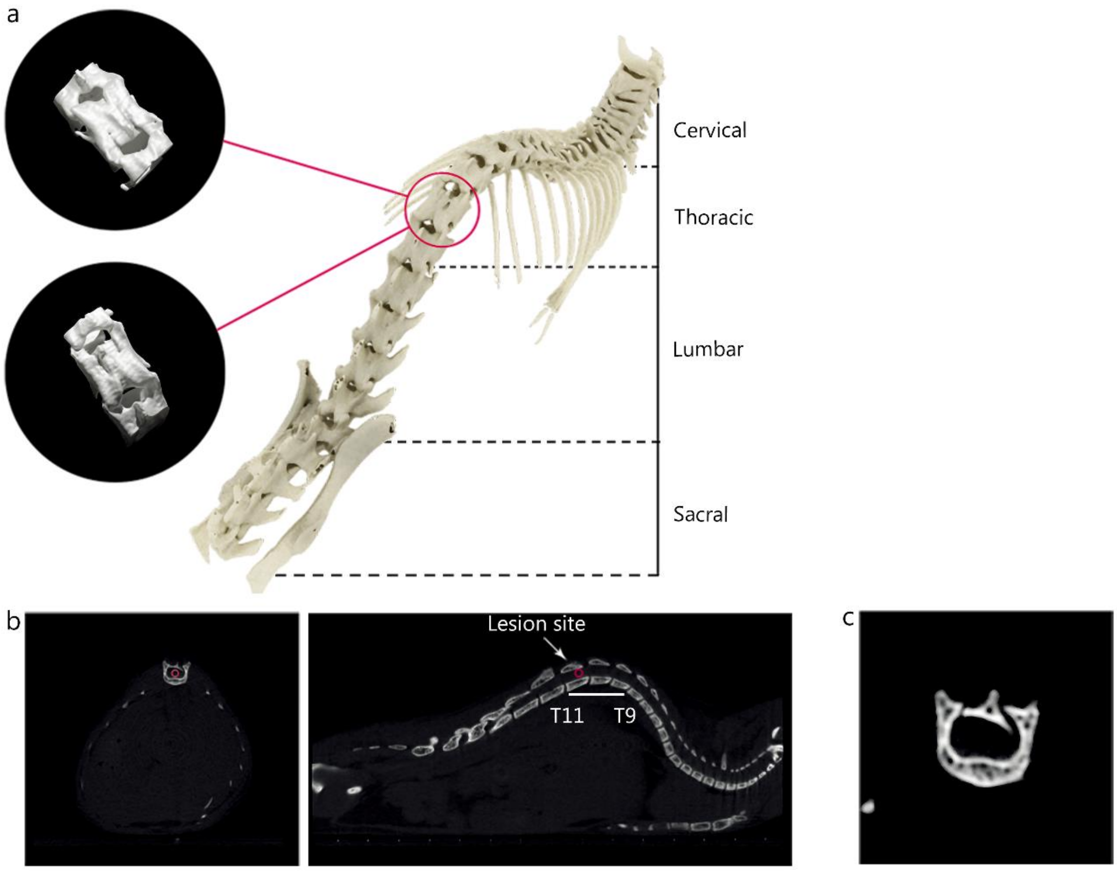

Fig. S1 Micro-computed tomography representative images of a contused spinal cord. **a** 3D reconstructed rendering of the thoracolumbar spine, showing the vertebral column and the site of injury (thoracic level). **b** Axial and sagittal views of the spinal region highlighting the area of contusion (between T9 and T11). **c** A particular axial section demonstrating the structural disruption at the lesion site

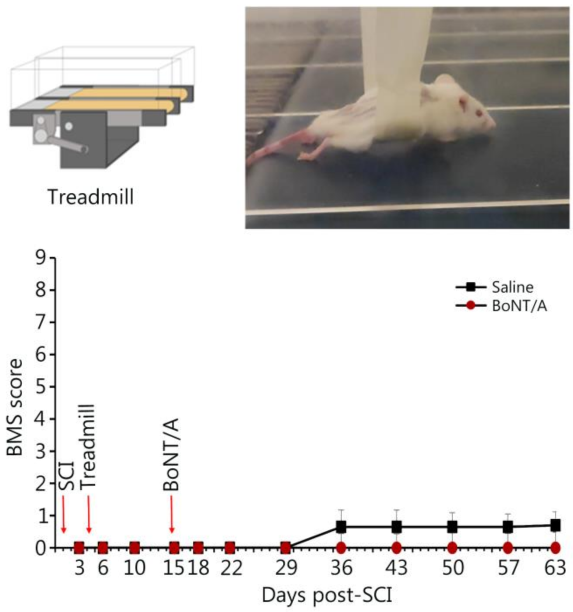

Fig. S2 Treadmill training protocol. To evaluate whether passive locomotor rehabilitation could facilitate recovery in the chronic phase, we tested a treadmill training protocol in severely injured mice. The rationale was to exploit residual spinal locomotor circuits (central pattern generators) that can be activated by repetitive stepping-like movements. Mice were placed on a motorized treadmill (model LE8710, PanLab, Cornella, Spain) starting 4 d post-injury, and the protocol was progressively intensified over the first week followed by maintenance training up to 63 d post-injury [4]. D4: 5 min at 5 cm/s+10 min at 10 cm/s; D5: 5 min at 10 cm/s+10 min at 15 cm/s; D6: 5 min at 15 cm/s+15 min at 20 cm/s; D7–63: 20 min at 20 cm/s, 5 d/week. Despite consistent application of the protocol, treadmill training did not improve locomotor outcomes in the severe spinal cord injury (SCI) group. As shown, Basso Mouse Scale (BMS) scores remained stable at 0–1 throughout the 60-day observation period, with no differences between BoNT/A- and saline-treated animals (Saline, n=3; BoNT/A, n=5). Based on this lack of efficacy, treadmill rehabilitation was not pursued further, and we focused on electrical muscle stimulation (EMS) as a non-invasive and translational strategy to prevent muscle atrophy and enhance responsiveness to BoNT/A treatment. BoNT/A. Botulinum neurotoxin type A

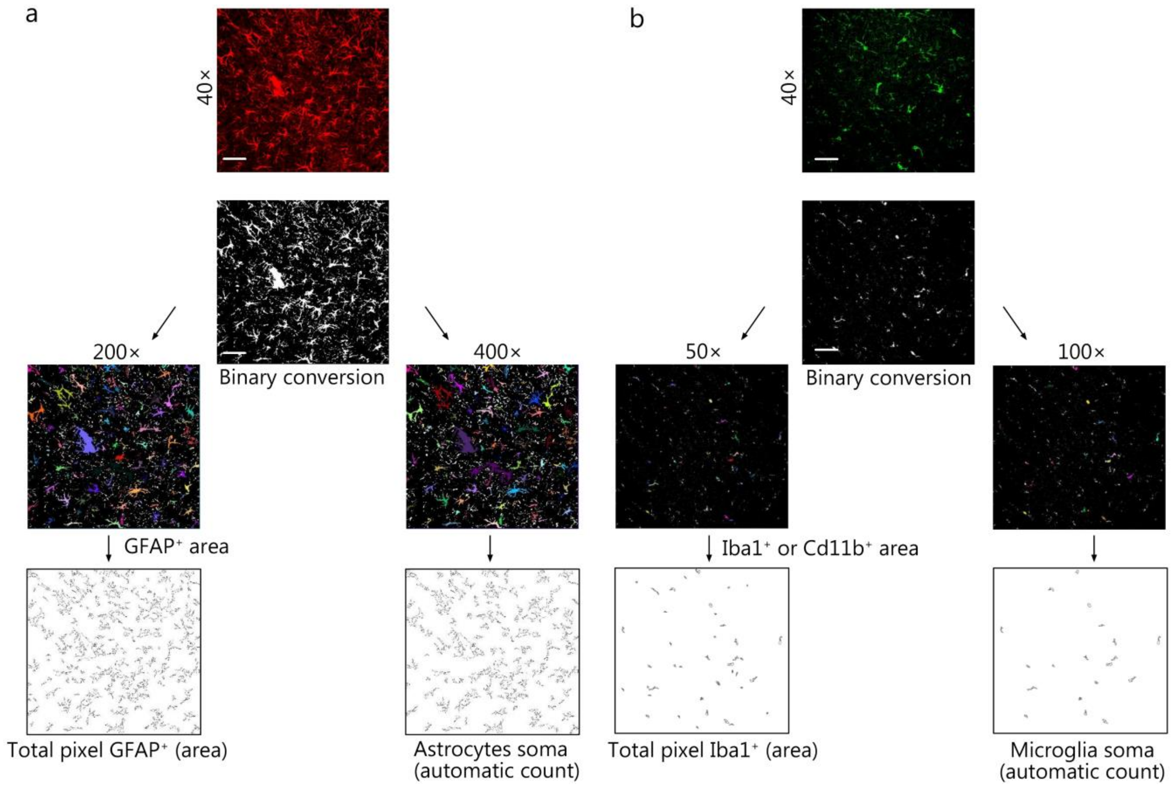

Fig. S3 Workflow for astrocyte and microglial quantification analysis. Representative sequence of image processing steps used for the automated quantification of GFAP$^+$ astrocytes (**a**) and Iba1$^+$/CD11b$^+$ microglia (**b**). **a** Astrocytes. Original confocal image of GFAP immunofluorescence (red) acquired from the spinal cord epicenter (40× magnification, scale bar=50 μm). The same image converted to 8-bit grayscale and binarized (white=GFAP$^+$ signal; black=background). Segmentation masks generated using the Analyze Particles function in ImageJ, applying two distinct size thresholds (200× for total GFAP$^+$ area; 400× for astrocytic somata). Each detected object is pseudo-colored for visualization. Binary outlines showing the individual astrocytic elements detected under each threshold condition. These steps enable automated quantification of both total GFAP$^+$ area and astrocyte number across spinal cord regions (epicenter, scar, perilesional areas). **b** Microglia. Original confocal image of Iba1 or CD11b immunofluorescence (green or red, depending on staining) acquired from the same anatomical regions (40× magnification, scale bar=50 μm). Image conversion to 8-bit grayscale and binarization to isolate microglia-positive signal. Segmentation of microglial elements using the Analyze Particles tool, with size thresholds adjusted to the smaller and more heterogeneous morphology of microglial cell bodies and processes [e.g., 50× for total Iba1$^+$/CD11b$^+$ area; (80–100)× for cell body counts]. Each segmented element is automatically assigned a unique color, highlighting the distribution and density of microglial structures. Binary outlines representing individual microglial elements under the two thresholding conditions. This workflow allows automated quantification of both the total microglia-positive area and the number of microglial cells, enabling comparisons across spinal cord regions (epicenter, scar, perilesional), and between experimental groups. GFAP. Glial

fibrillary acidic protein; Iba1. Ionized calcium binding adaptor moleclule 1; CD11b. Cluster of differentiation 11b

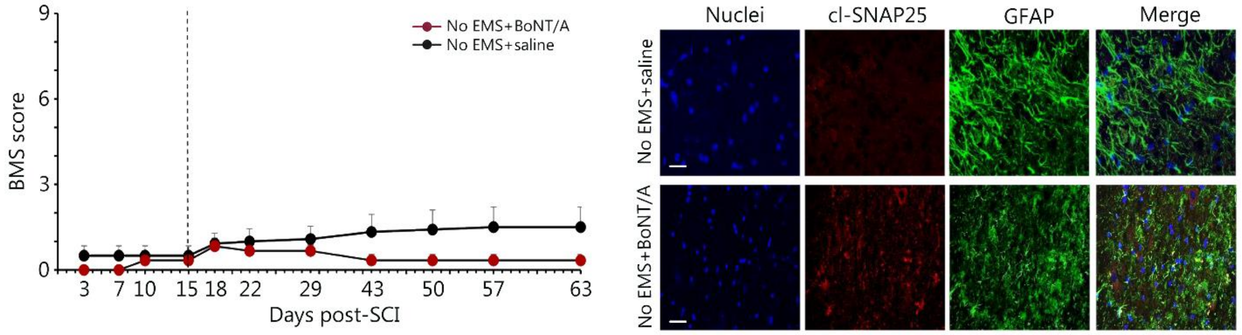

Fig. S4 Spinal administration of BoNT/A or saline in the chronic phase (15 d post-injury). **a** Motor function was assessed using the Basso Mouse Scale (BMS) following spinal cord injury (SCI). Dotted line indicates the time point of intrathecal injection of saline or BoNT/A. Only animals with severe injury (BMS score between 0 and 3) were included. No significant differences were observed between BoNT/A- and saline-treated animals (*n*=6; preliminary study). **b** Representative confocal images (40× magnification; scale bar=50 μm) of spinal cord tissue near the lesion site (T12–T13), collected 60 d post-injury. Sections were stained for nuclei (DAPI, blue), cleaved SNAP25 (cl-SNAP25, red), and glial fibrillary acidic protein (GFAP, green). In BoNT/A-treated animals, cl-SNAP25, absent in saline controls, is strongly expressed and partially colocalized with GFAP, indicating sustained toxin activity even 45 d after injection. A reduction in astrocyte activation is also apparent. BoNT/A. Botulinum neurotoxin type A; SNAP25. Synaptosomal-associated protein 25

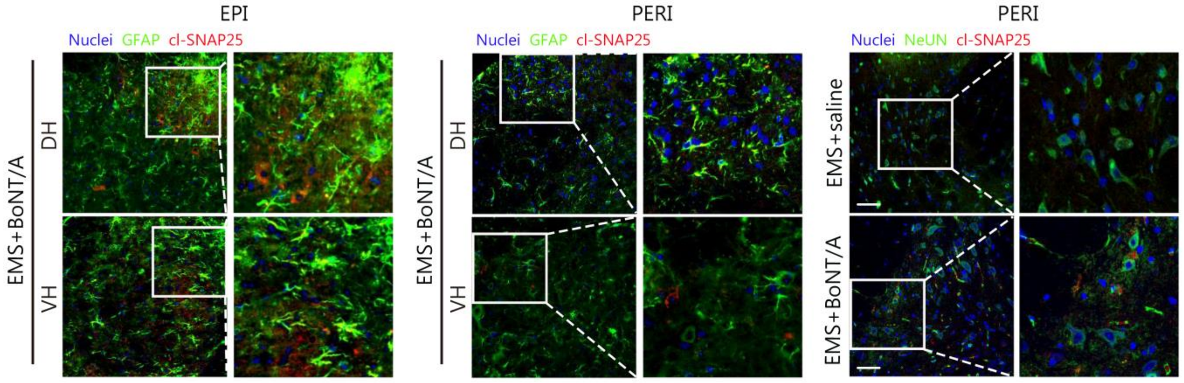

Fig. S5 Detection of cleaved SNAP25 (cl-SNAP25) in spinal cord 60 d post-injury. Representative immunofluorescence images (40×; scale bar=50 µm) showing cl-SNAP25 (red) in spinal cord sections collected from EPI (T9–T11) or /PERI regions (T7–T13) 60 d after injury. NeuN (green) was used to label neurons, and GFAP (green) to label astrocytes. Nuclei are counterstained with DAPI (blue). The inset on the right shows a saline-treated control section, while all other panels correspond to EMS+BoNT/A-treated animals. The white-boxed region is shown at 3× magnification on the right. The yellow colocalization signal highlights cl-SNAP25 within $GFAP^+$ or $NeuN^+$ cells, confirming the persistence of BoNT/A enzymatic activity in both neuronal and astrocytic compartments. The detection of cl-SNAP25 at thoracic levels distant from the lumbar injection site indicates a retrograde transport of the toxin and a long-lasting catalytic action up to 60 d post-administration. DH. Dorsal horn; VH. Ventral horn; BoNT/A. Botulinum neurotoxin type A; SNAP25. Synaptosomal-associated protein 25; NeuN. Neuron-specific nuclear protein; GFAP. Glial fibrillary acidic protein; DAPI. 4’,6-diamidino-2-phenylindole; EMS. Electrical muscle stimulation; EPI. Epicentre; PERI. Perilesional region

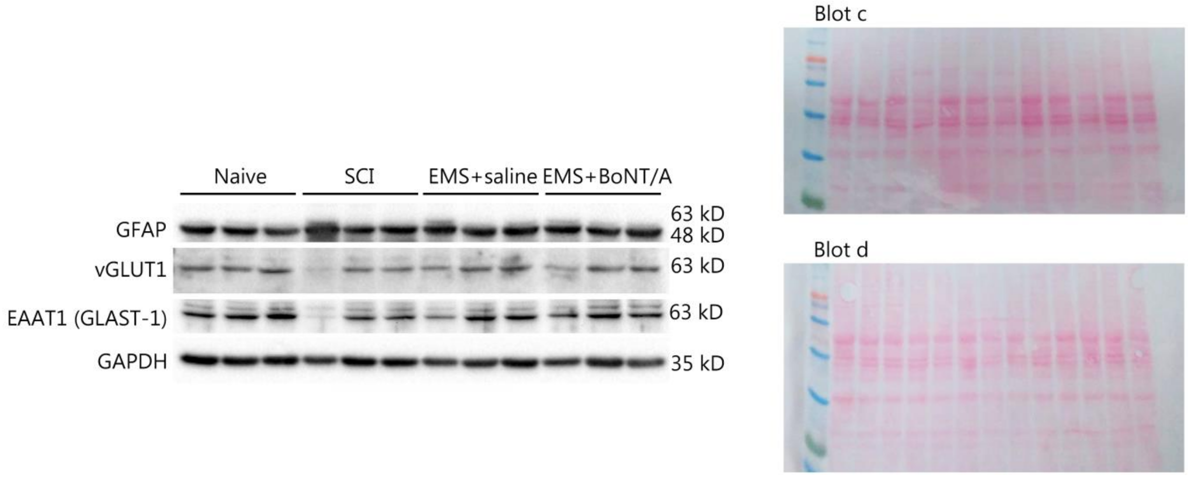

Fig. S6 Full panel of Western blotting experiments assessing GFAP, vGLUT1, EAAT1, GAPDH, and protein loading (Ponceau S staining – Blot c, d) in spinal cord tissue 60 d after SCI. Further blots from the same experimental groups (naive, SCI, EMS+saline, EMS+BoNT/A) used in **Fig. 4**, showing the expression of: GFAP, astrocytes marker; vGLUT1 and EAAT1 expression of glutamate transporters; GAPDH, used as reference proteins. Due to variable expression of housekeeping proteins across experimental conditions, normalization to GAPDH or β-actin was not applied. All samples refer to spinal cord lysates collected 60 d post-injury. BoNT/A. Botulinum neurotoxin type A; GFAP. Glial fibrillary acidic protein; vGLUT1. Vesicular glutamate transporter 1; EAAT1/GLAST-1. Excitatory amino acid transporter 1

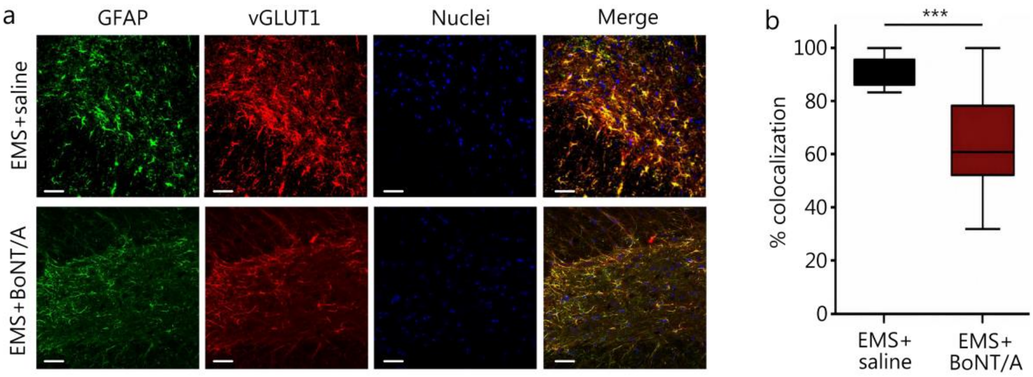

Fig. S7 Immunofluorescence for GFAP and vGLUT1 colocalization analysis. **a** Representative high-magnification confocal images (40×) from spinal cord sections collected 60 d after SCI, showing individual fluorescence channels for GFAP (red), vGLUT1 (green), and nuclei (DAPI, blue), as well as their merged image. Differential distribution and colocalization of astrocytic processes with the glutamate transporter across treatments. Scale bar=30 μm. **b** Box plot showing percentage of colocalization between GFAP (astrocytes) and vGLUT1 (excitatory presynaptic terminals) 60 d after SCI. BoNT/A significantly reduces GFAP-vGLUT1 colocalization compared to EMS+saline (unpaired *t*-test: t_{36}=5.13, P<0.0001; values represent mean±SEM). $^{***}P$<0.0001. BoNT/A. Botulinum neurotoxin type A; SCI. Spinal cord injury; GFAP. Glial fibrillary acidic protein; vGLUT1. Vesicular glutamate transporter 1; EAAT1. Excitatory amino acid transporter 1; DAPI. 4',6-diamidino-2-phenylindole

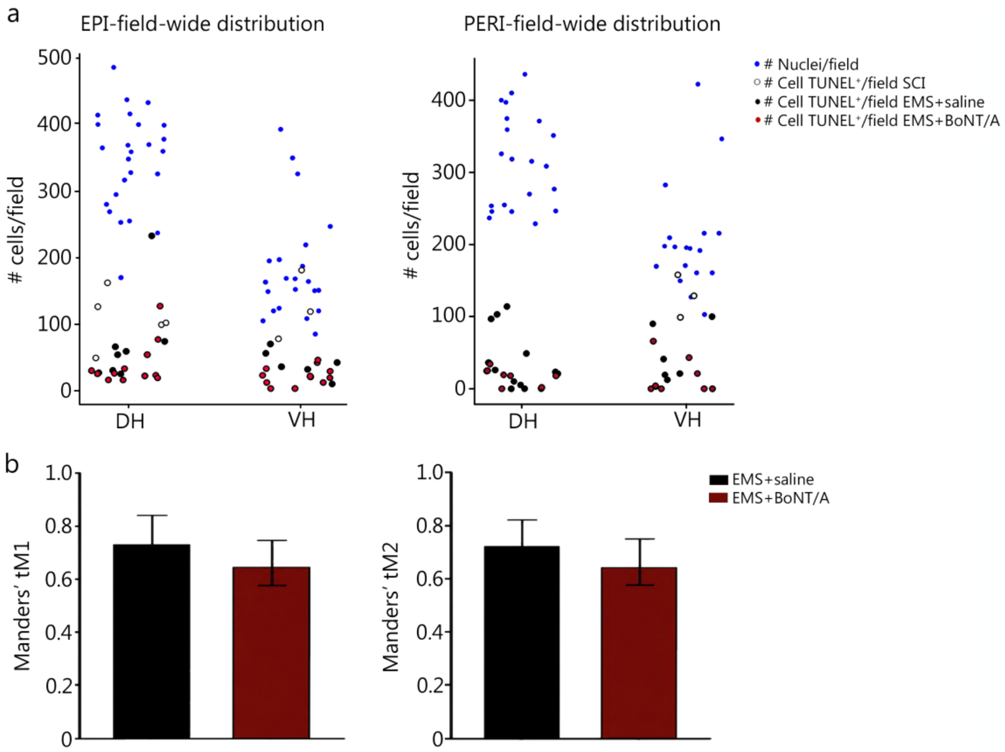

Fig. S8 Quantification of NeuN and TUNEL co-localization in spinal cord sections from EMS+saline and EMS+BoNT/A groups. **a** Nuclei (blue) and apoptotic cells from different groups distribution across the tissue. Individual values were plotted separately for the dorsal horn (DH) and ventral horn (VH). **b** Thresholded Manders' coefficients (tM1 and tM2), calculated above the automatic threshold for each channel, showed a similar trend but did not reach statistical significance. Data are presented as mean±SEM (*n*=3–4 animals/group, 7–14 slices/treatment). EMS. Electrical muscle stimulation; BoNT/A. Botulinum neurotoxin type A; SCI. Spinal cord injury; NeuN. Neuron-specific nuclear protein; TUNEL. Terminal deoxynucleotidyl transferase dUTP nick end labeling

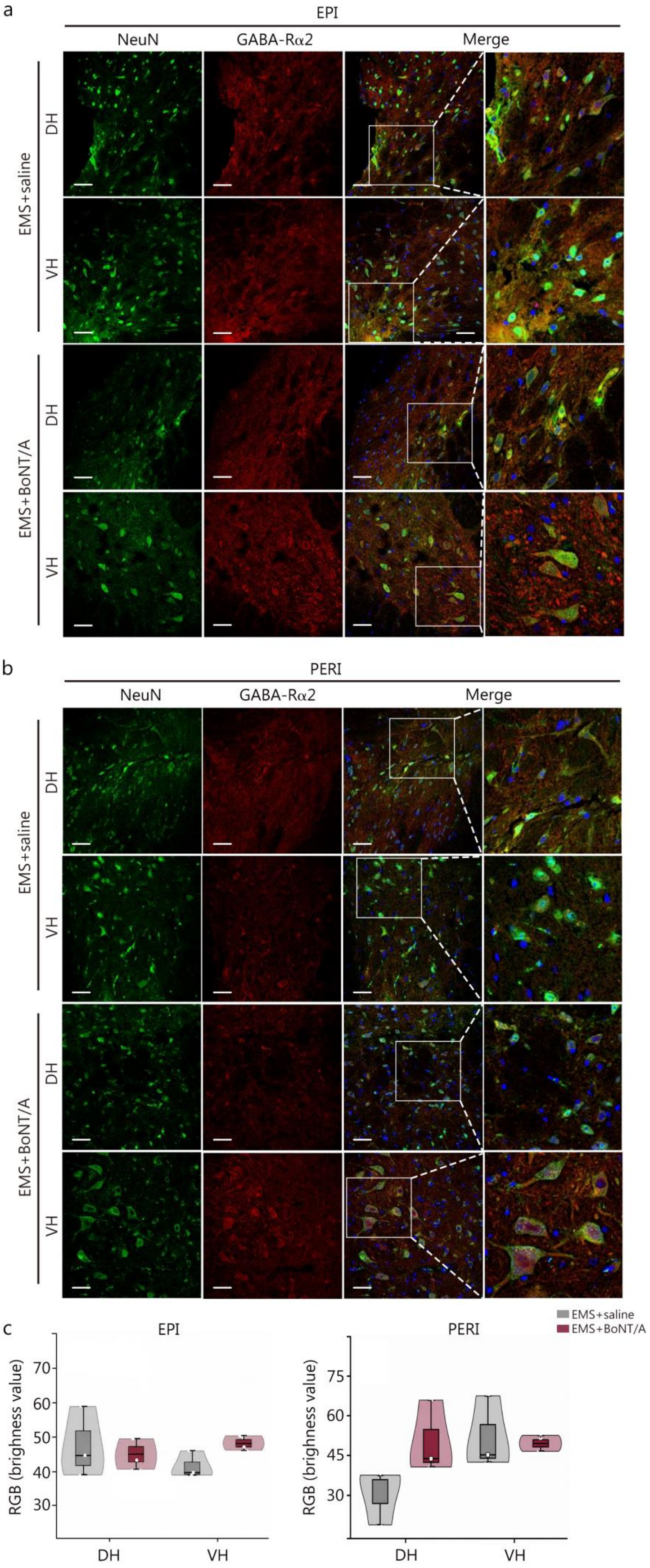

Fig. S9 Immunofluorescence analysis of GABA-A receptor subunit α2 (GABA-Rα2) in the spinal cord 60 d after SCI. **a, b** Localization and distribution of GABA-Rα2 immunoreactivity in the dorsal (DH) and ventral horns (VH) of the spinal cord from EMS+saline and EMS+BoNT/A treated animals. **a**

Representative image of the epicentral area (T9–T11), corresponding to the region directly affected by the trauma. **b** Representative confocal image of perilesional regions (rostral-caudal, within 2–3 mm from the impact zone; T7–T13 segments). Sections were co-stained for NeuN (neuronal marker, green), GABA-Rα2 (red), and nuclei (DAPI, blue). Images were acquired at 40× magnification; scale bar=50 µm. **c** Quantitative analysis of fluorescence intensity in DH and VH across epicentral and perilesional regions. Each point represents one animal (n=2–3). All slice-level values (one to two per animal for each area and DH/VH) were averaged per animal, which was considered the experimental unit. Boxplots display median (IQR), and violin plots illustrate data distribution. Because of the small sample size (n<5), non-parametric descriptive statistics were applied. Kruskal-Wallis and Scheirer-Ray-Hare tests did not reveal significant differences between treatments or areas (P>0.05), indicating that GABA-A receptor expression patterns are comparable between EMS+saline and EMS+BoNT/A groups. EMS. Electrical muscle stimulation; BoNT/A. Botulinum neurotoxin type A; SCI. Spinal cord injury; NMDA: N-methyl-D-aspartate; GABA-A: Gamma-aminobutyric acid type A receptor; EPI. Epicentre; PERI. Perilesional region; IQR. Interquartile range; DAPI. 4',6-diamidino-2-phenylindole

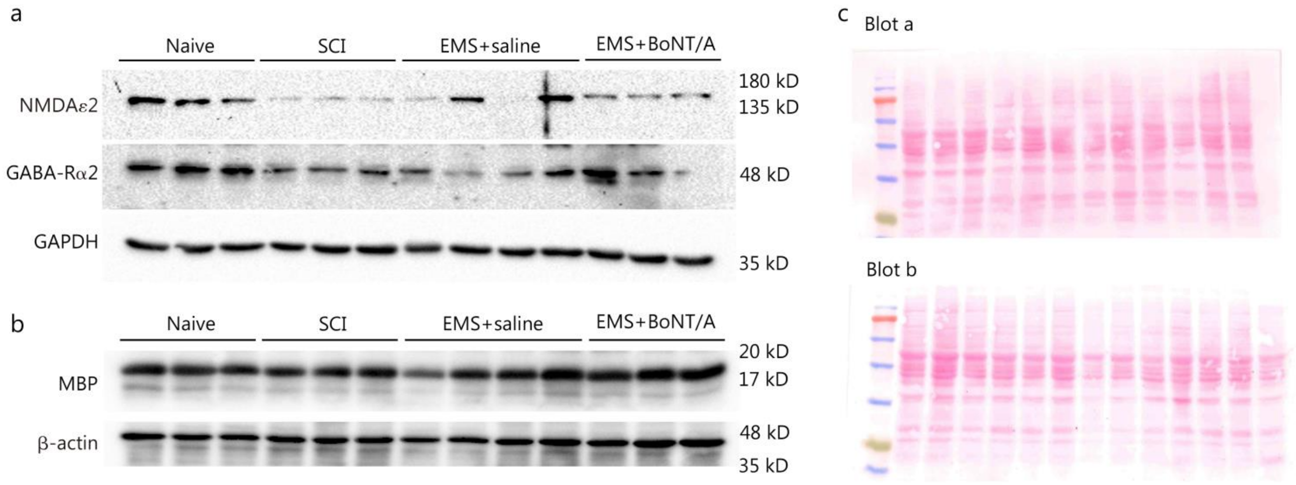

Fig. S10 Full panel of Western blotting experiments assessing receptor subunits, myelin integrity, and loading controls in spinal cord tissue 60 d after SCI. **a** Further blots from the same experimental groups (naive, SCI, EMS+saline, EMS+BoNT/A) used in **Fig. 7**, showing the expression of: NMDA receptor subunit ε2 (NMDAε2) and GABA-A receptor subunit α2 (GABA-Rα2), associated with excitatory and inhibitory neurotransmission. **b** Myelin basic protein (MBP), a marker of myelin integrity; GAPDH and β-actin, used as reference proteins. Due to variable expression of housekeeping proteins across experimental conditions (see **Fig. 7** legend), normalization to GAPDH or β-actin was not applied. **c** Ponceau S staining used to verify total protein loading and transfer efficiency across lanes. Upper membrane (Blot a) corresponds to the Western blotting experiments shown in panel **a** (NMDAε2, GABA-Rα2 and GAPDH). Lower membrane (Blot b) corresponds to the Western blotting experiments shown in panel **b** (MBP and β-actin). All samples refer to spinal cord lysates collected 60 d post-injury. EMS. Electrical muscle stimulation; BoNT/A. Botulinum neurotoxin type A; SCI. Spinal cord injury

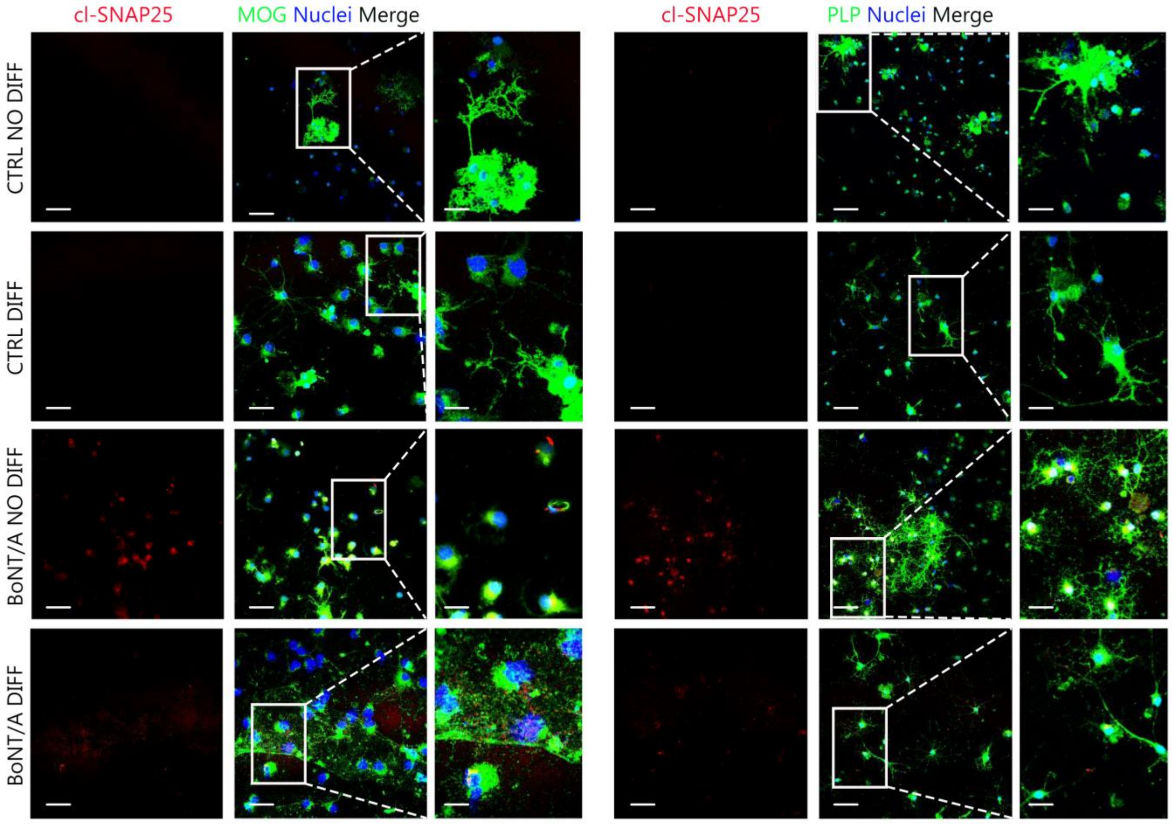

Fig. S11 BoNT/A internalization in oligodendroglial cells demonstrated by colocalization with cl-SNAP25. Representative confocal images (40×, scale bar=50 μm) of primary oligodendrocyte cultures stained with antibodies against cl-SNAP25 (red), the oligodendrocyte maturation markers myelin oligodendrocyte glycoprotein (MOG, green, left panel) or proteolipid protein (PLP, green, right panel), and nuclear counterstain (DAPI, blue). The white-boxed region is shown at 3× magnification on the right. BoNT/A uptake and functional enzymatic activity were evaluated by immunolabeling for cl-SNAP25, a specific marker of BoNT/A-mediated SNAP25 cleavage. Images show partial or complete colocalization between cl-SNAP25 and MOG^{+} or PLP^{+} cells. BoNT/A. Botulinum neurotoxin type A; cl-SNAP25. Cleaved synaptosomal-associated protein 25

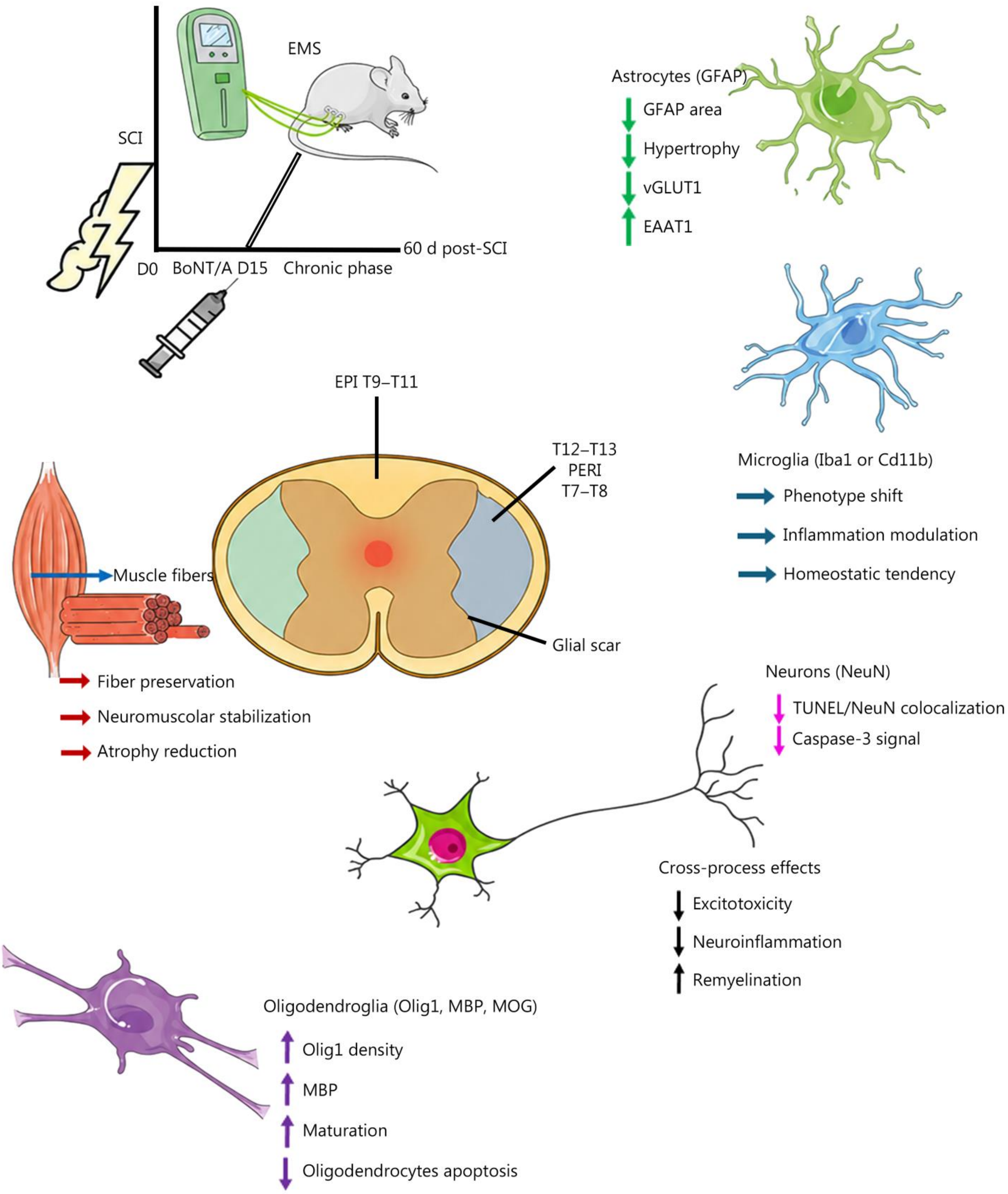

Fig. S12 Schematic representation illustrates the experimental design and the multi-level effects of combined EMS+BoNT/A treatment administered during the chronic phase of spinal cord injury (SCI). At 60 d post-injury, mice underwent an EMS rehabilitation protocol, with BoNT/A delivered at day 15 of the chronic phase. Histological and molecular analyses were performed in two spinal cord regions, epicenter (EPI: T9–T11) and perilesion (PERI: T8–T7/T12–T13), as well as in hindlimb muscles. The combined treatment modulated several cellular targets. Astrocytes (GFAP): reduced hypertrophy and GFAP area, decreased vGLUT1, and increased EAAT1 expression. Microglia (Iba1 or Cd11b): shift toward less reactive phenotypes, modulation of inflammatory profiles, and increased homeostatic

features. Muscle fibers: preservation of fiber size, reduced atrophy, and stabilization of neuromuscular junctions. Oligodendroglia (Olig1/MBP/MOG): increased density, enhanced maturation, and improved myelin-related markers and reduced apoptosis. Neurons (NeuN): reduced TUNEL/NeuN colocalization and decreased caspase-3 activation. Cross-process analyses indicate reduced excitotoxicity, attenuated neuroinflammation, and enhanced remyelination, supporting the therapeutic potential of EMS+BoNT/A in chronic SCI. EMS. Electrical muscle stimulation; BoNT/A. Botulinum neurotoxin type A; GFAP. Glial fibrillary acidic protein; Iba1. Ionized calcium binding adaptor moleclule 1; vGLUT1. Vesicular glutamate transporter 1; EAAT1. Excitatory amino acid transporter 1; NeuN. Neuron-specific nuclear protein; TUNEL. Terminal deoxynucleotidyl transferase dUTP nick end labeling; MBP. Myelin basic protein; MOG. Myelin oligodendrocyte glycoprotein; Olig1. Oligodendrocyte lineage transcription factor 1

References

1. Vacca V, Madaro L, De Angelis F, Proietti D, Cobianchi S, Orsini T, *et al.* Revealing the therapeutic potential of botulinum neurotoxin type A in counteracting paralysis and neuropathic pain in spinally injured mice. Toxins (Basel). 2020;12(8):491. https://doi.org/10.3390/toxins12080491.
2. Marinelli S, Vacca V, De Angelis F, Pieroni L, Orsini T, Parisi C, *et al*. Innovative mouse model mimicking human-like features of spinal cord injury: efficacy of docosahexaenoic acid on acute and chronic phases. Sci Rep. 2019;9(1):8883. https://doi.org/10.1038/s41598-019-45037-x.
3. Barrière G, Leblond H, Provencher J, Rossignol S. Prominent role of the spinal central pattern generator in the recovery of locomotion after partial spinal cord injuries. J Neurosci. 2008;28(15):3976. https://doi.org/10.1523/JNEUROSCI.5692-07.2008.
4. Cobianchi S, Marinelli S, Florenzano F, Pavone F, Luvisetto S. Short- but not long-lasting treadmill running reduces allodynia and improves functional recovery after peripheral nerve injury. Neuroscience. 2010;168(1):273. https://doi.org/10.1016/j.neuroscience.2010.03.035.
